# Cell identity and filament architecture shape intercellular communication in the terminal heterocystous cyanobacterium *Richelia rhizosoleniae* SC01

**DOI:** 10.1128/aem.00935-26

**Published:** 2026-09-10

**Authors:** Sepehr Bardi, Mercedes Nieves-Morión, Rachel A. Foster

**Affiliations:** 1 Department of Ecology, Environment and Plant Sciences, Stockholm University 7675 , Stockholm, Sweden; 2 Instituto de Bioquímica Vegetal y Fotosíntesis, CSIC and Universidad de Sevilla, Seville, Spain; Georgia Institute of Technology, Atlanta, Georgia, USA

**Keywords:** heterocyst-forming cyanobacteria, intercellular communication, septal junctions, nanopores, *Richelia rhizosoleniae *SC01, *Anabaena *sp. PCC 7120, FRAP, FLIP

## Abstract

Intercellular communication is essential for metabolite exchange and cellular coordination in filamentous cyanobacteria, yet how filament organization influences this process remains poorly understood, particularly in terminal heterocyst-forming strains. Here, we compared the facultative symbiont *Richelia rhizosoleniae* SC01 (hereafter *Richelia*; terminal heterocysts) with the free-living *Anabaena* sp. PCC 7120 (hereafter *Anabaena* sp.; intercalary heterocysts) and a septal junction mutant of *Anabaena* with impaired intercellular communication (CSVT22, ∆*fraC*-∆*fraD*), using fluorescence recovery after photobleaching (FRAP) and fluorescence loss in photobleaching (FLIP). *Richelia* SC01 and *Anabaena* showed similar recovery (*R*) rates, indicating comparable average molecular exchange through septal junctions, with no significant correlation between *R* and filament length. By contrast, CSVT22 showed reduced recovery and accelerated, irregular decay, indicative of both disrupted septal connectivity and altered regulation of septal junctions, resulting in heterogeneous and less efficient intercellular exchange. Differences in fluorescence decay speed across cell types and strains suggest that cell identity and filament organization, including filament taper, shape patterns of intercellular exchange. FLIP analyses showed that *Anabaena* consistently exhibited greater exchange asymmetry, whereas *Richelia* SC01 vegetative cells displayed comparatively more symmetric exchange, despite pronounced filament tapering and terminal heterocyst organization. Transmission electron microscopy further showed that septal nanopores in *Richelia* SC01 are similar in size to those of *Anabaena* but exhibited marked variation in number per septal disk, suggesting a potential structural basis for variation in intercellular exchange patterns. Collectively, our findings show that combining FRAP and FLIP provides a quantitative framework to dissect the kinetics and spatial organization of intercellular exchange in filamentous cyanobacteria.

**IMPORTANCE:**

Filamentous cyanobacteria rely on intercellular exchange through septal junctions to coordinate metabolism and development, yet the principles governing multicellular communication in bacterial filaments remain poorly understood. Here, we show that intercellular exchange emerges from the interplay between septal junction properties, cell identity, and filament architecture, generating distinct communication patterns across cyanobacterial lifestyles. Notably, despite its pronounced filament tapering and terminal heterocyst organization, *Richelia* SC01 exhibited comparatively symmetric exchange, demonstrating that filament polarity alone does not determine communication patterns. Our findings reveal that cyanobacterial multicellularity arises from the integration of structural organization and cell-specific properties, providing a framework for understanding coordinated physiology in filamentous cyanobacteria, including symbiotic and nitrogen-fixing systems.

## INTRODUCTION

Multicellularity and cellular differentiation likely originated with filamentous microbes (1–4). One of the foundational characteristics of multicellular organisms is the presence of structures that enable coordinated activity between cells through intercellular communication. Among prokaryotes, filamentous cyanobacteria stand out as some of the earliest examples of multicellularity and cellular differentiation (2, 4), where cytoplasmic bridges play crucial roles in coordinating cellular activity (5). In eukaryotes, we see phylogenetically widespread examples of such structures enabling cytoplasmic continuity: “plasmodesmata” in land plants; “gap junctions” in animals; and “septal pores” in fungi (6).

Filamentous cyanobacteria that differentiate into distinct cell types, e.g., heterocyst-forming cyanobacteria, represent ideal model systems to study cooperation between two distinct cell types: vegetative cells and heterocysts. Under nitrogen-limiting (diazotrophic) conditions, heterocysts differentiate to perform N_2_ fixation. Depending on the strain, heterocysts can be single and positioned terminally or in set intervals along the filament (intercalary) (7). Consequently, the proportion of heterocysts along the filament (heterocyst frequency) is influenced by environmental conditions and varies among cyanobacteria strains. In particular, cyanobacteria that form symbiotic relationships with terrestrial plants can have 3- to 10-fold (up to 60% in land plant symbioses) higher heterocyst frequency than their free-living counterparts (8). Vegetative cells are always present and function to reduce CO_2_ through oxygenic photosynthesis and provide energy for N_2_ fixation. Metabolite exchange between the two cell types, where nitrogen-enriched metabolites from heterocysts (9–12) are exchanged for photosynthates from the vegetative cells (mainly as sucrose) (13–16), is well documented under diazotrophic conditions. Thus, both cell types are mutually dependent and require intercellular communication for metabolite exchange and growth of the whole filament.

In the model heterocyst-forming cyanobacterium *Anabaena* sp. PCC 7210, rapid intercellular exchange of metabolites and signaling molecules is enabled through cytoplasmic connections between adjacent cells, formed by septal junctions. The septal junctions in filamentous cyanobacteria traverse the septal peptidoglycan layer through perforations called nanopores to establish a cytoplasmic continuity between neighboring cells (17–19). Several proteins, including FraC and FraD (5, 20, 21) and SepJ (also known as FraG) (22), function in the formation of the septal junctions. Nanopores have been visualized by transmission electron microscopy (TEM) of purified peptidoglycan murein sacculi in several different filamentous cyanobacteria, including *Anabaena* sp. PCC 7210, and are considered key components enabling intercellular exchange (23, 24). In *Anabaena* sp. PCC 7120, the formation of nanopores requires AmiC-type amidases (AmiC1, Alr0092; AmiC2, Alr0093) that perforate the septal peptidoglycan, while AmiC3 (All1140) influences nanopore width, and a LytM-factor regulator activates AmiC1 to control the process; inactivation of any of these proteins results in reduced nanopore abundance and impaired intercellular molecular exchange (23, 25–27). Thus, filamentous heterocyst-forming cyanobacteria provide an ideal model system to investigate the links between cell differentiation, multicellular organization, and intercellular communication.

Intercellular molecular exchange in filamentous cyanobacteria has been commonly studied using fluorescence recovery after photobleaching (FRAP) analysis. FRAP relies on sequesterable fluorescent tracers (e.g., calcein, 5-carboxyfluorescein, and esculin), photobleaching, and real-time live cell imaging (28–30). In FRAP, the recovery rate of fluorescence in the bleached cell directly reflects the diffusion rate and serves as a measure of intercellular communication rates. It assumes that intercellular communication is through simple diffusion (28–30). While the influence of nanopore size and abundance per disk on recovery rates has been characterized in vegetative cells of *Anabaena* sp. PCC 7210 (23), the influence of other characteristics at the filament level, such as filament length, cell size, and heterocyst position, on intercellular diffusion dynamics remains largely unexplored. A second complementary live cell imaging technique that also relies on retainable fluorophores, fluorescence loss in photobleaching (FLIP), can be used to quantify the intercellular exchange along filaments by measuring the spreading decay of fluorescence to the surrounding cells due to continuous bleaching of one cell. However, FLIP has so far only been used to study intercellular communication through the continuous periplasm of cyanobacterial filaments (31). FLIP can therefore enable the quantification of the rate and symmetry of intercellular exchange along cyanobacterial filaments, and, when combined with FRAP, provides a comprehensive framework to resolve both local cell-to-cell exchange and filament-scale transport dynamics.

Here, we characterized the kinetics and structural aspects of intercellular communication in the marine filamentous facultative cyanobacterial symbiont *Richelia rhizosoleniae* strain SC01 (hereafter *Richelia* SC01) that differentiates terminal heterocysts and can form symbioses with chain-forming diatoms (32). There are two other *Richelia* strains (ReuHH01 and RintRC01) which live in symbiosis with diatoms, but as endobionts. The filaments of *Richelia* SC01 exhibit a tapering to that of one endobiont, ReuHH01, with vegetative cell diameters decreasing with distance from the terminal heterocyst (33, 34). In symbiosis, *Richelia* strains (including SC01) are the primary source of nitrogen to their respective host (35, 36), and strategically, *Richelia* SC01 attaches externally to the diatom with its heterocyst. Thus, determining *Richelia’s* capacity for intercellular communication and its spatial organization within filaments is important to understand the cellular basis of symbiont-host interaction. We further compared intercellular communication in *Richelia* SC01 with the model cyanobacterium *Anabaena* sp. PCC 7120 (hereafter *Anabaena*) and the *Anabaena* double ∆*fraC-*∆*fraD* mutant (hereafter CSVT22) using FRAP and FLIP methods. The double mutant, CSVT22, shows a phenotype resembling *Richelia* SC01: short filaments and terminal heterocysts, and is largely impaired in intercellular communication. Given the importance of septal structures in intercellular communication, we additionally imaged and characterized the nanopores of *Richelia* SC01. The combined use of FRAP and FLIP revealed that intercellular exchange in *Richelia* SC01 occurs at rates comparable to *Anabaena* yet differs in spatial asymmetry and cell type-specific dynamics, revealing heterogeneous patterns of exchange. Our study demonstrates that filament architecture and lifestyle shape the organization but not the overall capacity of intercellular communication in filamentous cyanobacteria.

## RESULTS

### Morphological comparison of cyanobacterial strains

To establish structural characteristics relevant to the FRAP and FLIP analyses, we measured filament length, heterocyst frequency, heterocyst position, and cell cross-sectional area for all strains grown under the same diazotrophic lab conditions (Fig. 1A). We additionally collected images of wild *R. rhizosoleniae* from a confocal image archive of plankton samples from *Tara Oceans* (37) and previously reported (38, 39). The latter images represent the symbiotic state for *Richelia* SC01, as all images show *R. rhizosoleniae* living in symbioses with its preferred diatom host *Chaetoceros* sp. (32).

**Fig 1:**
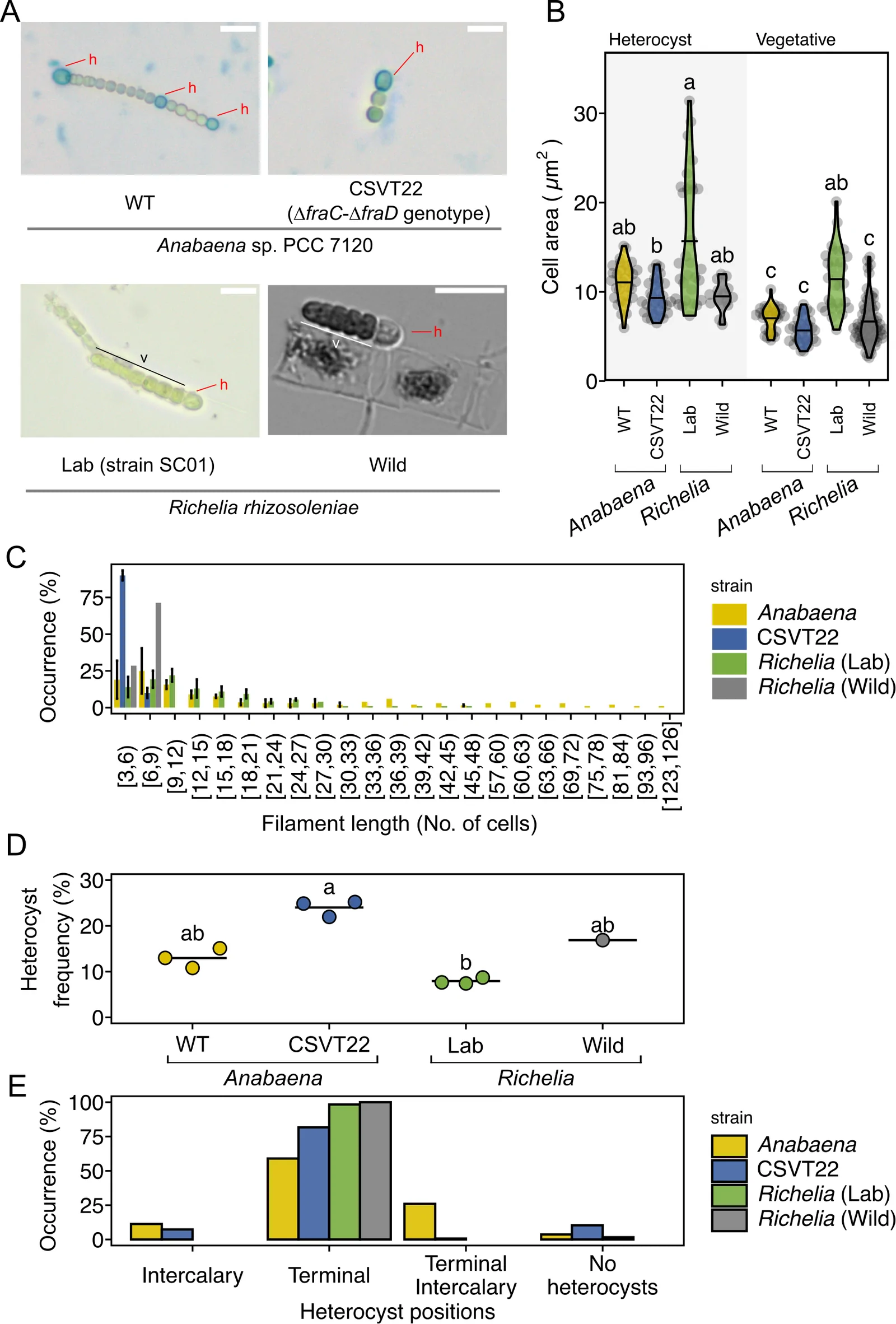
Comparison of cell morphological features in cyanobacterial strains used in this study. (**A**) Brightfield micrographs of *Anabaena* sp. PCC 7120, *Anabaena* ∆*fraC*-∆*fraD* double mutant (CSVT22), and *Richelia rhizosoleniae* from this study (lab strain *Richelia* SC01) and wild populations (symbiotic with diatom of genus *Chaetoceros* sp.). The CSVT22 filaments selected for live-cell imaging in this study comprised two vegetative cells and a single terminal heterocyst. Heterocysts of *Anabaena* and CSVT22 were stained using alcian blue. Micrograph of the wild population *R. rhizosoleniae* was obtained from a confocal image archive of plankton samples from *Tara Oceans* (37) and previously reported (38, 39). Scale bars are 10 µm. v, vegetative cells; h, heterocyst. (**B**) Cross-sectional cell area of heterocysts and vegetative cells for each respective cyanobacteria strain. Groups are significantly different if marked by different letters according to a Kruskal-Wallis/Dunn test (*H*[7] = 221.3, *P* < 0.001, *n* = 263 cells). (**C**) Proportions of filament lengths (cells per filament). *Anabaena* and CSVT22 cultures were monitored 48 h after growth in diazotrophic conditions. Error bars indicate the standard deviation of the mean of triplicates within each bin. Bins between 3 and 24 in filament size had data from each triplicate, while those above either had data from one or two triplicates. For *Richelia* (wild), 14 filaments in total were counted. (**D**) Heterocyst frequency was calculated as the number of heterocysts divided by the total number of cells in each replicate for each strain. Each point represents the mean of a replicate, and the bar represents the mean of all replicates. Groups are significantly different if marked with different letters according to a Kruskal-Wallis/Dunn test (*H*[3] = 8.3, *P* = 0.039, *n* = 10 replicates). (**E**) Proportion of filaments with heterocysts in different positions: intercalary, terminal, both terminal and intercalary, or no heterocysts.

Cell cross-sectional area varied significantly within strains. In *Anabaena* strains, heterocysts were consistently larger than vegetative cells, whereas no significant differences between cell types were observed in *Richelia* SC01 (Fig. 1B). Between *Richelia* strains, vegetative cells of SC01 were 1.7-fold larger than those of wild *Richelia*, while heterocyst size did not differ significantly.

Of the laboratory strains, *Richelia* SC01 showed the largest cell areas overall, with heterocysts and vegetative cells being 1.4- to 1.7-fold and 1.6- to 2-fold larger, respectively, than those of *Anabaena* and CSVT22. In terms of cell shape, *Richelia* strains exhibited lower circularity, indicating more elliptical elongated cells than *Anabaena* strains in both cell types (Fig. S1). Within strains, only *Richelia* SC01 showed cell type-specific differences in shape, with vegetative cells presenting more elongated morphologies than heterocysts (Fig. S1). The previously reported taper in the *Richelia* SC01 filaments was also noted in both the lab and wild strains. In contrast, all *Anabaena* strains did not differ significantly in cell shape between cell types. Accordingly, the longest cell diameter in *Richelia* SC01 was approximately 2.6-fold larger than in wild *Richelia* for both cell types, and vegetative cells were also larger than those of *Anabaena* (~1.4- to 1.5-fold), whereas heterocyst diameter did not differ significantly among strains. Altogether, only *Anabaena* strains showed significant cell type differences in shape and size, with heterocysts being larger and less elliptical than vegetative cells.

The cell density per filament varied with strains. For filaments of similar length, the number of cells differed between *Richelia* SC01 and wild *Richelia* (12 ± 7 and 6 ± 1 cells per filament, respectively; Fig. 1C). For *Anabaena*, we observed high variability in filament length after handling (16 ± 17 cells per filament), whereas for the CSVT22 mutant, we observed the shortest filaments (4 ± 1 cells per filament). The high variation in *Anabaena* is likely a result of handling, which resulted in high fragmentation of filaments. *Richelia* SC01 and *Anabaena* had the largest ranges of filament length, ranging from 3–45 cells and 3–123 cells, respectively.

Heterocyst frequency (number of heterocysts/total number of cells) was highest in CSVT22 after 48 h in diazotrophic conditions (24.0% ± 1.8%), comparable to wild *Richelia* (16.9%) and *Anabaena* (13.0% ± 2.1%), and lowest in *Richelia* SC01 (7.9% ± 0.7%; Fig. 1D). Heterocyst positioning within filaments was strain specific. Both *Richelia* strains had exclusively terminal heterocysts. In the CSVT22 mutant, most filaments contained terminal heterocysts (82%), whereas 10% lacked heterocysts and 7% had intercalary heterocysts. In *Anabaena*, potential filament breakage during handling resulted in a mixed distribution of heterocyst positions, with filaments containing terminal (59%), both terminal and intercalary (26%), or exclusively intercalary heterocysts (11%; Fig. 1E).

Overall, the cyanobacterial strains analyzed exhibit substantial morphological variation in cell types and filament architecture, providing the structural framework required for subsequent analyses to understand intercellular exchange.

### Intercellular communication in cyanobacterial filaments differs between strains and cell types

Cells (heterocyst or vegetative cell) labeled with 5-CF were successfully photobleached in all three cyanobacteria strains, allowing measurement of fluorescence recovery by FRAP experiments (Fig. 2A). Although 5-CF labeling indicated viable cells, two distinct behaviors were observed independently of strain and cell type after photobleaching: some cells showed fluorescence recovery, while others remained bleached, indicating the absence of lateral communication with their neighboring cells (Fig. 2B). The time required to reach the 90% saturation recovery after photobleaching differed among all strains as well as between cell types in *Richelia* SC01 and CSVT22 (Fig. 2B). For example, 90% saturation in vegetative cells and heterocysts was reached approximately after 15 s and 21 s, respectively, in *Richelia* SC01; 19 s and 20 s, respectively, in *Anabaena*; and 22 s and 23 s, respectively, in CSVT22 (Fig. 2B).

**Fig 2:**
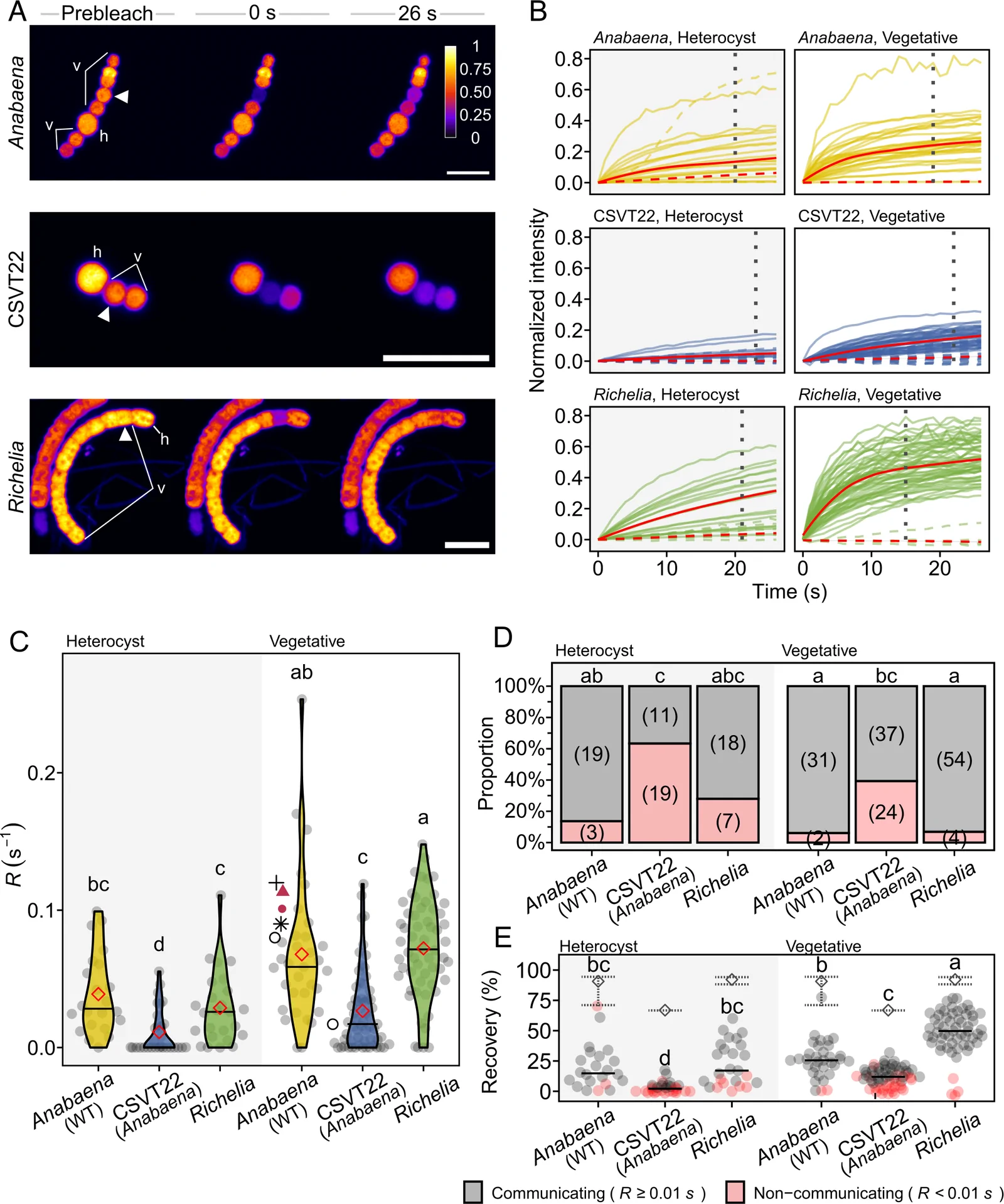
Summary of parameters from FRAP experiments. (**A**) Representative time-lapse confocal laser scanning microscopy micrographs from the FRAP experiments (30 s, includes prebleach frames) of *Anabaena*, CSVT22, and *Richelia* SC01 under experimental conditions for bleaching a vegetative cell (white arrow). “h” designates heterocyst, and “v” designates vegetative cell. Color scale bar indicates normalized fluorescence intensity. Scale bar is 10 µm. (**B**) Normalized FRAP curves for heterocysts and vegetative cells. Fluorescence intensity in bleached cells was range normalized, i.e., background-corrected intensity was divided by the mean intensity of three prebleach images. Solid lines are communicating cells, and dashed lines are non-communicating cells (based on *R* values; see legend in panel D). The red lines are local regressions (LOESS) showing the population averages. Vertical dotted lines indicate the time at which 90% of the recovery plateau is reached for each group (communicating cells only). (**C**) Estimated recovery rates based on recovery curves in panel B. The central lines indicate the median. Groups are significantly different if marked with different letters according to a Kruskal-Wallis/Dunn test (*H*[5] = 80.74, *P* < 0.001, *n* = 229 cells). Groups are not significantly different if marked with the same letter. Both the communicating and non-communicating populations were included in the analysis. Red diamonds indicate the mean. The symbols indicate the mean of *R* values from previous studies, as follows: plus symbol, (23); triangle, (40); filled circle, (41); asterisk, (5); open circle, (30). The color of the symbols indicates which medium and amended *N* condition was used (pink = BG11_0_ with no amended *N*, black = BG11 and amended *N*). Note that the data from Bornikoel et al. (23) and Nürnberg et al. (30) are based on mean values, while the others are based on median values where non-communicating cells were included for all except Arévalo and Flores (40). Data from FRAP experiments correspond to at least three independent biological replicates per strain. (**D**) Proportions of non-communicating cells for each strain and cell type. Groups are significantly different if marked with different letters according to a pairwise proportion test (χ^2^[5] = 45.94, *P* < 0.001, *n* = 229 cells). Numbers in parentheses indicate the number of observations. (**E**) Percentage of fluorescence recovery for strains and cell types. Black points indicate communicating cells (*R* ≥ 0.01 s^−1^), and red points indicate non-communicating cells (*R* < 0.01 s^−1^). The hollow diamonds represent the mean maximum theoretical recovery, calculated as the total number of cells minus the one bleached cell, divided by the total number of cells in the filament. The dotted error bars indicate the standard deviation in theoretical recovery based on filament length. The mean maximum theoretical recovery values were 90.6% (10.6 cells), 66.7% (3 cells), and 92.1% (12.7 cells) for *Anabaena*, CSVT22, and *Richelia* SC01, respectively. Groups are significantly different if marked with different letters according to a Dunn test (*H*[5] = 120.88, *P* < 0.001, *n* = 229 cells). Groups are not significantly different if marked with the same letter. The central line is the median.

We measured the average fluorescence recovery for vegetative cells and heterocysts, defined as the percentage of the prebleach fluorescence intensity reached after 30 s of recovery, to quantify the extent of fluorescent 5-CF available for intercellular exchange within the whole filament. We took into consideration the overall filament length (Fig. 1C) to calculate the theoretical maximum fluorescence intensity recovery percentage (*F*_max_) if all the 5-CF taken up would be freely available and all intercellular communication opened. Consequently, *F*_max_ was lower for the short filament of CSVT22 than in the longer filaments of *Anabaena* and *Richelia* SC01 (Fig. 2E).

We observed a significant cell type-specific difference in average fluorescence recovery for *Richelia* SC01 and CSVT22, but not for *Anabaena*: heterocysts exhibited a lower average recovery than vegetative cells (Fig. 2E). In *Richelia* SC01, average recovery was 23% ± 18% in heterocysts compared to 49% ± 18% in vegetative cells (*F*_max_ = 92%), while in CSVT22 values were 3% ± 4% and 12% ± 8%, respectively (*F*_max_ = 67%). In vegetative cells, average fluorescence recovery was highest in *Richelia* SC01 (49% ± 18%), intermediate in *Anabaena* (26% ± 15%; *F*_max_ = 91%), and lowest in CSVT22 (12% ± 8%; Fig. 2B and E).

We therefore first focused on characterizing the capacity of intercellular exchange in single cell types within filaments. The recovery rate constant (*R*; s^−1^), derived from a mathematical fit of range-normalized FRAP recovery profiles (all exhibiting similar AIC range), was used to compare strains and cell types (Fig. 2C, Table 1). Our results were consistent with previous work under similar diazotrophic conditions on vegetative cells of *Anabaena* and CSVT22 (Fig. 2C). Independent of the recovery profiles, we found that the overall distribution of *R* values was similar between *Richelia* SC01 and *Anabaena*, and both strains had significantly higher and larger ranges in *R* values than CSVT22. When comparing cell types, *Richelia* SC01 and CSVT22 showed lower *R* values and range in heterocysts, whereas *Anabaena* showed significant differences in *R* range but not values between cell types (Fig. 2C, Table 1). Overall, our results indicate that each individual cell type can vary its intercellular communication capacity with its neighbors within the filament. However, it remains unclear whether the differences in this capacity are due to filaments, strains, cell types, and/or position within the filament.

**TABLE 1:** Summary of recovery rates, *R*, for heterocysts and vegetative cells of *Anabaena*, the CSVT22 mutant, and *Richelia* SC01^*a*^

	Mean *R* ± SD (*n*) (s^−1^)		
	Comm. and non-comm. cells	Comm. cells (*R* ≥ 0.01 s^−1^)	Non-comm. cells (*R* < 0.01 s^−1^)
Vegetative			
*Anabaena*	0.068 ± 0.052 (33)	0.072 ± 0.051 (31)	0.000 ± 0.000 (2)
CSVT22	0.027 + 0.029 (61)	0.042 ± 0.028 (37)	0.003 ± 0.003 (24)
*Richelia* SC01	0.072 ± 0.036 (58)	0.078 ± 0.031 (54)	0.0002 ± 0.0004 (4)
Heterocyst			
*Anabaena*	0.039 ± 0.029 (22)	0.044 ± 0.028 (19)	0.004 ± 0.004 (3)
CSVT22	0.011 ± 0.016 (30)	0.030 ± 0.014 (11)	0.0007 ± 0.002 (19)
*Richelia* SC01	0.029 ± 0.027 (25)	0.039 ± 0.025 (18)	0.003 ± 0.004 (7)

^*a*^ Data are presented and separated according to the criterion for non-communication (*R* < 0.01 s^−1^). The total number of observations (*n*) was derived from at least three separate experimental occasions.

We then classified cell types according to their *R* values, and cells with *R* < 0.01 s^−1^ were classified as non-communicating, as previously defined (5, 42). We measured the proportion of non-communicating cell types in each strain: *Richelia* SC01 exhibited similar proportions of non-communicating heterocysts and vegetative cells (28% and 7%, respectively) as *Anabaena* (14% and 6%, respectively), contrasting with CSVT22 (63% and 39%, respectively; Fig. 2D). Both cell types in *Anabaena* and only vegetative cells in *Richelia* SC01 had significantly lower proportions of non-communicating cells compared to CSVT22 (Fig. 2D). Taken together, our results suggest that each cell in a filament, independent of the strain, can autonomously regulate its communication through cytoplasmic connections with its neighboring cells.

Our FRAP data demonstrated that the isolated cell behavior depended on filaments. We then assessed the performed linear regressions to compare how filament length affected intercellular communication parameters (fluorescence recovery and recovery rate; Table 2). In all cases, correlations were weak (*R*^2^ < 0.5) and/or not significant, indicating that intercellular communication did not systematically change with filament length. These results indicate that the high variability in *Anabaena* filament length did not systematically influence fluorescence recovery measurements despite its effect on the theoretical maximum recovery (*F*_max_). Overall, independent of cell type and strains, our results highlight that the intercellular communication capacity of each cell in a filament is controlled at the single cell level.

**TABLE 2:** Linear regression results for *Anabaena* and *Richelia* where filament length was defined as a predictor variable and either fluorescence recovery or recovery rate (*R*) was defined as a response variable^*a*^

Response variable	Strain	Cell type	*n*	Filament length (mean ± SD)	Slope (± SE)	Intercept (± SE)	*R*²	*P*-value
Fluorescence recovery (%)	*Anabaena*	Heterocyst	22	12 ± 9	0.0131 ± 0.0034	0.04 ± 0.05	0.426	<0.001
		Vegetative	33	10 ± 5	0.0137 ± 0.0044	0.13 ± 0.05	0.233	<0.01
	*Richelia*	Heterocyst	15	16 ± 6	−0.0062 ± 0.0084	0.36 ± 0.14	0.041	0.472
		Vegetative	31	11 ± 2	0.0201 ± 0.0114	0.27 ± 0.13	0.097	0.0875
Recovery rate, *R* (s⁻¹)	*Anabaena*	Heterocyst	22	12 ± 9	−0.0006 ± 0.0007	0.05 ± 0.01	0.041	0.364
		Vegetative	33	10 ± 5	0.0003 ± 0.0017	0.07 ± 0.02	0.001	0.88
	*Richelia*	Heterocyst	15	16 ± 6	−0.0005 ± 0.0011	0.04 ± 0.02	0.016	0.65
		Vegetative	31	11 ± 2	0.0026 ± 0.0024	0.04 ± 0.03	0.041	0.277

^*a*^ Only filaments which could be observed in whole were included. The same samples (filaments) were used for both response variables. Filament length was measured as the number of cells per filament.

### Decay kinetics, decay mechanisms, and patterns of intercellular exchange in FLIP assays vary strongly with strain and cell type

Given the results of the FRAP assays, we then used FLIP experiments (Fig. S2) to evaluate whether (i) the position of cells in a filament and/or (ii) the spatial distribution of intercellular exchange between neighboring cells are differently affected. To assess the FLIP results, we fitted our normalized fluorescence decay profiles onto a stretched exponential (StrExp) model (equation 4), from which we extracted for every cell on the filament, differentially distant from the continuously bleached cell, the following parameters: the time constant (τ or Tau) describing the speed of fluorescence decay, the stretching exponent (*h*) indicating hetero- to homogeneity extent of decay (estimated by the curvature extent of fitted curve), and the time-dependent rate coefficient [*k*(*t*)] describing how the loss of fluorescence evolves over time (43).

We first compared the characteristic decay time (τ) of cells in a filament at several distances from the bleached cell, counted in cell distance (two- and five-cell distances; see the supplemental methods). When comparing the average τ of cells within a two-cell distance from the bleached cell, CSVT22 filaments bleached in a vegetative cell decayed significantly faster (τ = 58.5 ± 37.4 s, *n* = 60) than *Anabaena* vegetative cells (τ = 96.4 ± 64.6 s, *n* = 25) or *Richelia* SC01 heterocysts (τ = 134 ± 110 s, *n* = 32; Fig. 3A). In the five-cell range (restricted to *Anabaena* and *Richelia* SC01), *Richelia* SC01 filaments bleached in a vegetative cell decayed significantly faster (τ = 79.0 ± 30.2 s, *n* = 15) than in *Richelia* SC01 heterocyst (τ = 165 ± 113 s, *n* = 32) or *Anabaena* vegetative cell (τ = 122 s ±66.3, *n* = 25; Fig. 3B).

**Fig 3:**
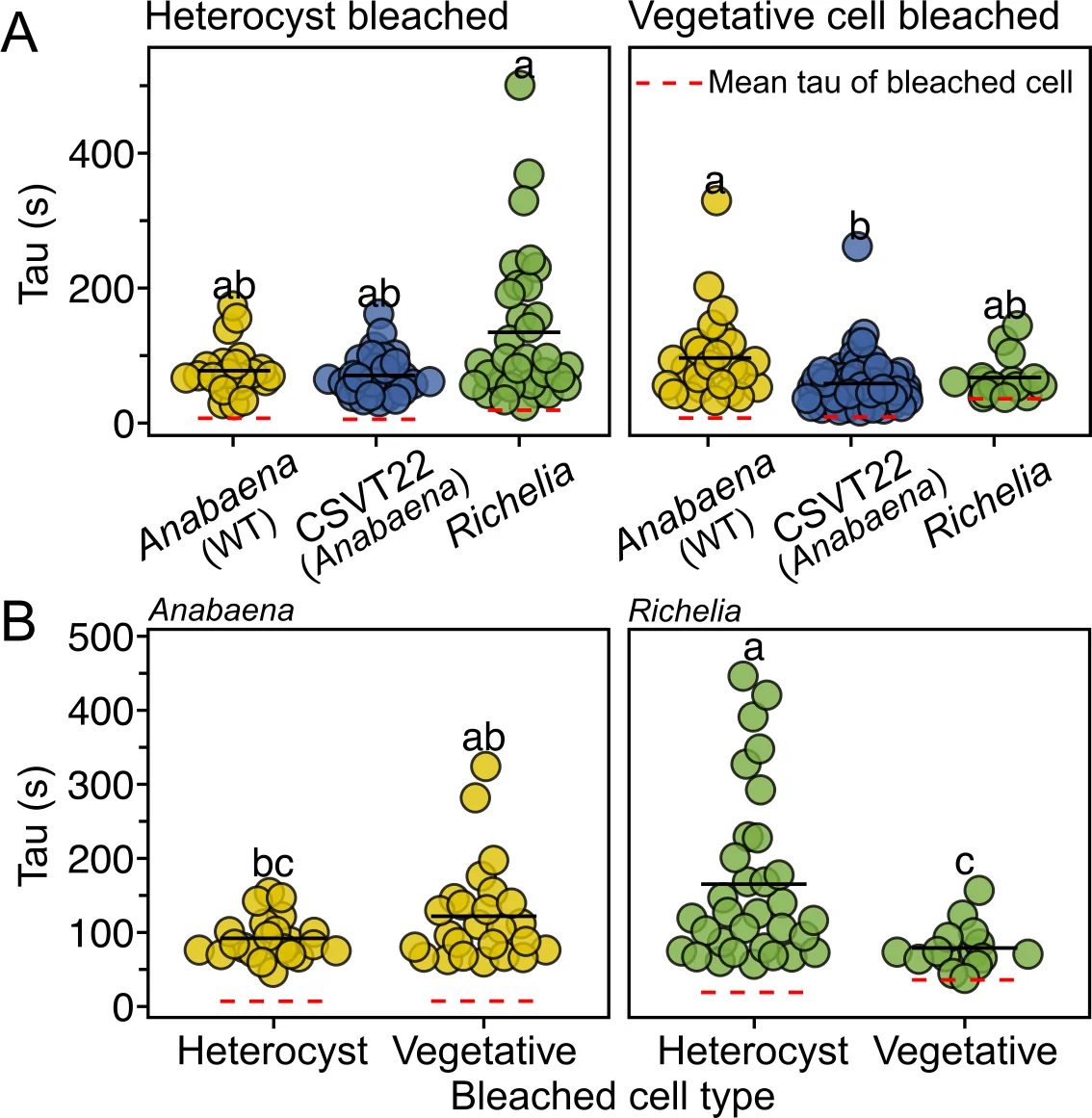
Comparisons of time constant (Tau) distributions. (**A**) The average time constant for cells within a two-cell distance from the bleached cell. The central lines indicate the mean. The red dashed lines indicate the mean time constant of the bleached cell itself, provided as a reference point for the decay kinetics of neighboring cells. Groups are significantly different if marked with different letters according to a Kruskal-Wallis/Dunn test (*H*[5] = 26.50, *P* < 0.001, *n* = 184 filaments). (**B**) The average time constant for *Anabaena* and *Richelia* SC01 cells within a five-cell distance from the bleached cell. The central lines indicate the mean. Groups are significantly different if marked with different letters according to a Kruskal-Wallis/Dunn test (*H*[3] =14.1, *P* < 0.01, *n* = 94 filaments). CSVT22 was not included because filaments were too short. Groups are not significantly different if marked with the same letter.

To elucidate the factors driving fluorescence decay kinetics, we applied a linear mixed-effects model (LMM; Fig. 4A; Fig. S3) with log-transformed τ as the dependent variable. Among multiple variables tested, including strain, bleached cell type, cell type, heterocyst frequency, filament length, and model root mean squared error (RMSE)—the distance from the bleached cell was one of the strongest predictors of τ, consistent with diffusion-mediated decay along filaments (Fig. 4B). Importantly, bleaching of *Richelia* SC01 vegetative cells produced a significant negative effect on τ indicating faster decay, whereas *Richelia* SC01 filaments overall, including heterocyst, showed a significant positive effect on τ (slower decay). In contrast, heterocyst frequency, cell type, and filament length had no significant effect, highlighting that spatial position and cell type of bleaching are the primary determinants of filament decay dynamics (Fig. 4B).

**Fig 4:**
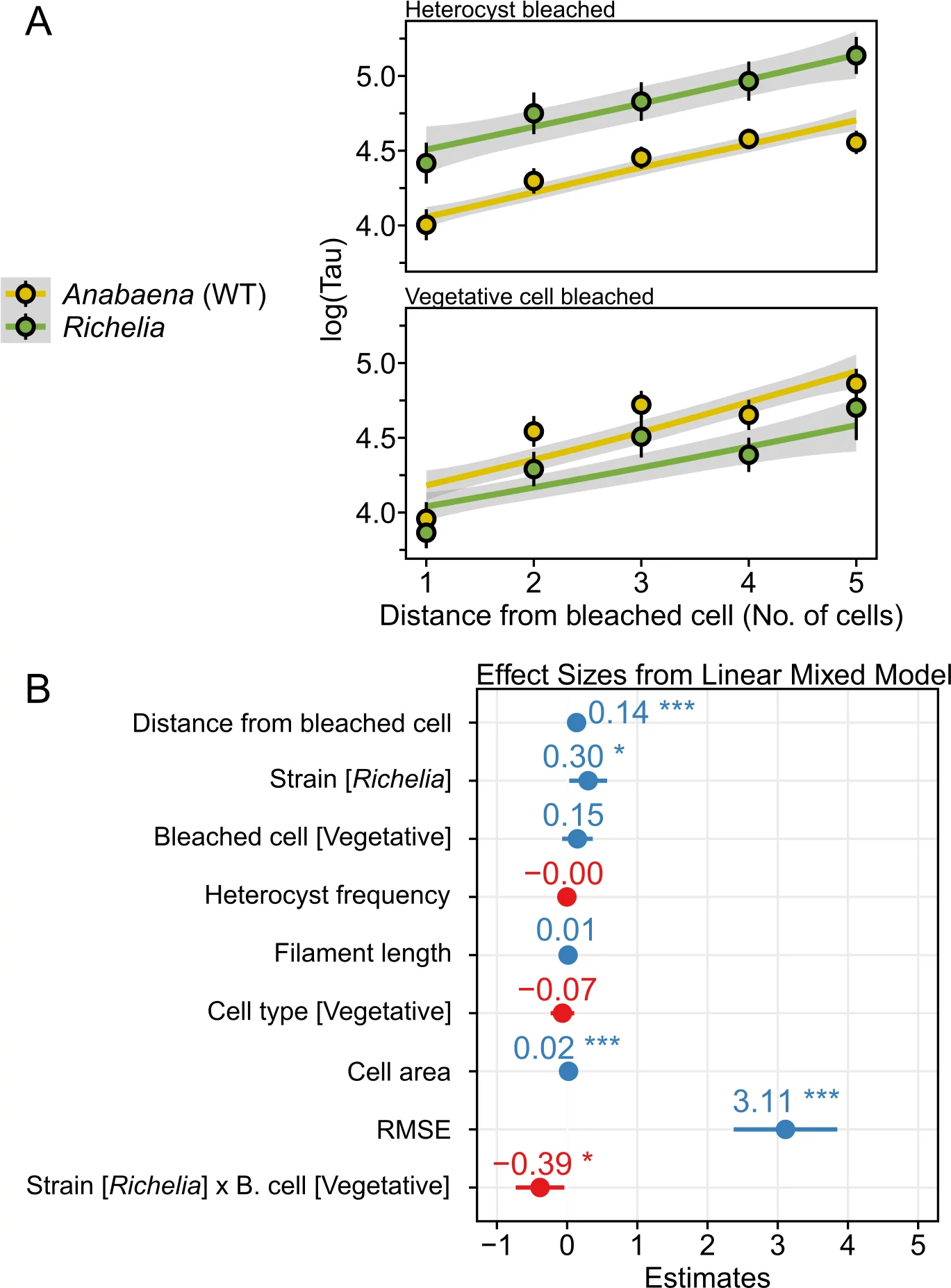
Linear mixed-effects modeling (LMM) of FLIP decay kinetics in filamentous cyanobacteria. (**A**) Relationship between the time constant of fluorescence decay [log(Tau)] and the distance from the bleached cell, shown separately for heterocyst-bleached (top) and vegetative-bleached (bottom) conditions. Each line represents a species-specific smoothed fit (generalized additive models, GAM), with shaded areas indicating 95% confidence intervals and points showing mean ± SE. A positive gradient of tau with distance is consistent with diffusion-limited transport mechanisms (43). (**B**) Effect sizes from the LMM explaining variability in fluorescence decay kinetics (log-transformed Tau) in FLIP experiments. Fixed effects included distance from the bleached cell, species (*Richelia rhizosoleniae* SC01 vs *Anabaena* sp. PCC 7120), bleached cell type (heterocyst vs vegetative), cell type (measurement cell), total cell number per filament, heterocyst frequency, cell area, and StrExp model RMSE. Random intercepts were included for filament identity (sample name). Points represent estimated effect sizes with 95% confidence intervals. Significant negative effects indicate factors associated with faster fluorescence decay (lower Tau), whereas positive effects indicate slower decay (higher Tau). * *P* < 0.05, ** *P* < 0.01, and *** *P* < 0.001.

To further explore the mechanisms underlying the distance-dependent and cell type differences in fluorescence decay revealed by the kinetic analysis of τ, we classified the nature of fluorescence loss using a qualitative framework adapted from Wüstner et al. (43), based on three variables: τ, the characteristic decay time reflecting the overall speed of fluorescence loss, *h*, the shape parameter indicating the curvature of the decay curve, with lower values (<0.5) corresponding to irregular or disconnected decays, and *k*(*t*), the instantaneous decay rate describing how the loss of fluorescence evolves over time (Table 3; Fig. S4). Each decay curve was assigned to one of six categories: diffusion-limited (inside or outside the ROI), binding/barrier-limited, or diffusion-limited, mono-exponential (bleach-limited), very low *h* (disconnected/ambiguous), or unclassified. As expected for diffusion-mediated decay, *h* values decreased (curves became more compressed) with increasing distance, resulting in a higher proportion of cells with low *h* (<0.5; Fig. 5). In *Richelia* SC01, heterocyst bleaching produced a higher fraction of very low *h* cells than bleaching of vegetative cells at both two-cell (39% and 6%, respectively) and five-cell (61% and 28%, respectively) distances (Fig. 5), indicating more irregular or disconnected decay in heterocysts. By contrast, *Anabaena* and CSVT22 showed no significant cell type-dependent differences, and CSVT22 filaments contained a smaller proportion of very low *h* cells overall, reflecting more uniform fluorescence decay (Fig. 5).

**TABLE 3:** Definitions of fluorescence loss (FL) categories in this study, grounded in the StrExp model parameters [*h*, τ, and *k*(*t*)], adapted from Wüstner et al. (43)^*a*^

Category (FL-loss type)	Stretching parameter (*h*)	*k*(*t*) behavior (slope)	Interpretation/explanation
Diffusion-limited (inside ROI)	1 < *h* < 2	Decreasing *k*(*t*) over time	Rapid bleaching inside ROI dominated by diffusion limitations—fluorophore replenishment slows down over time
Diffusion-limited (outside ROI)	0.5 < *h* < 0.8	Increasing *k*(*t*) over time	Compressed decay outside ROI; delayed response consistent with diffusion limitation at distance
Binding/barrier-limited or diffusion-limited	0.8 ≤ *h* < 1	Increasing *k*(*t*) over time	Fluorophore mobility hindered by binding or structural barriers causing delayed decay
Mono-exponential decay (bleach-limited)	*h* ≈ 1	Constant over time and equal to 1/τ	Approx. mono-exponential decay determined by the bleach rate
Very low *h* (disconnected/ambiguous)	*h* < 0.5	Variable *k*(*t*)	Indicative of severely limited or ambiguous intercellular connectivity, due to barriers or distance from the bleached cell
Unclassified	Any other combination	–^*b*^	Patterns that do not fit other categories

^*a*^ τ is the characteristic decay time reflecting the overall speed of fluorescence loss. *h* is the shape parameter indicating the curvature of the decay curve, with lower values (<0.5) corresponding to irregular or disconnected decays. *k*(*t*) is the instantaneous decay rate describing how the loss of fluorescence evolves over time.

^*b*^ – indicates that there are no specific patterns of the *k*-slope that would describe the unclassified category.

**Fig 5:**
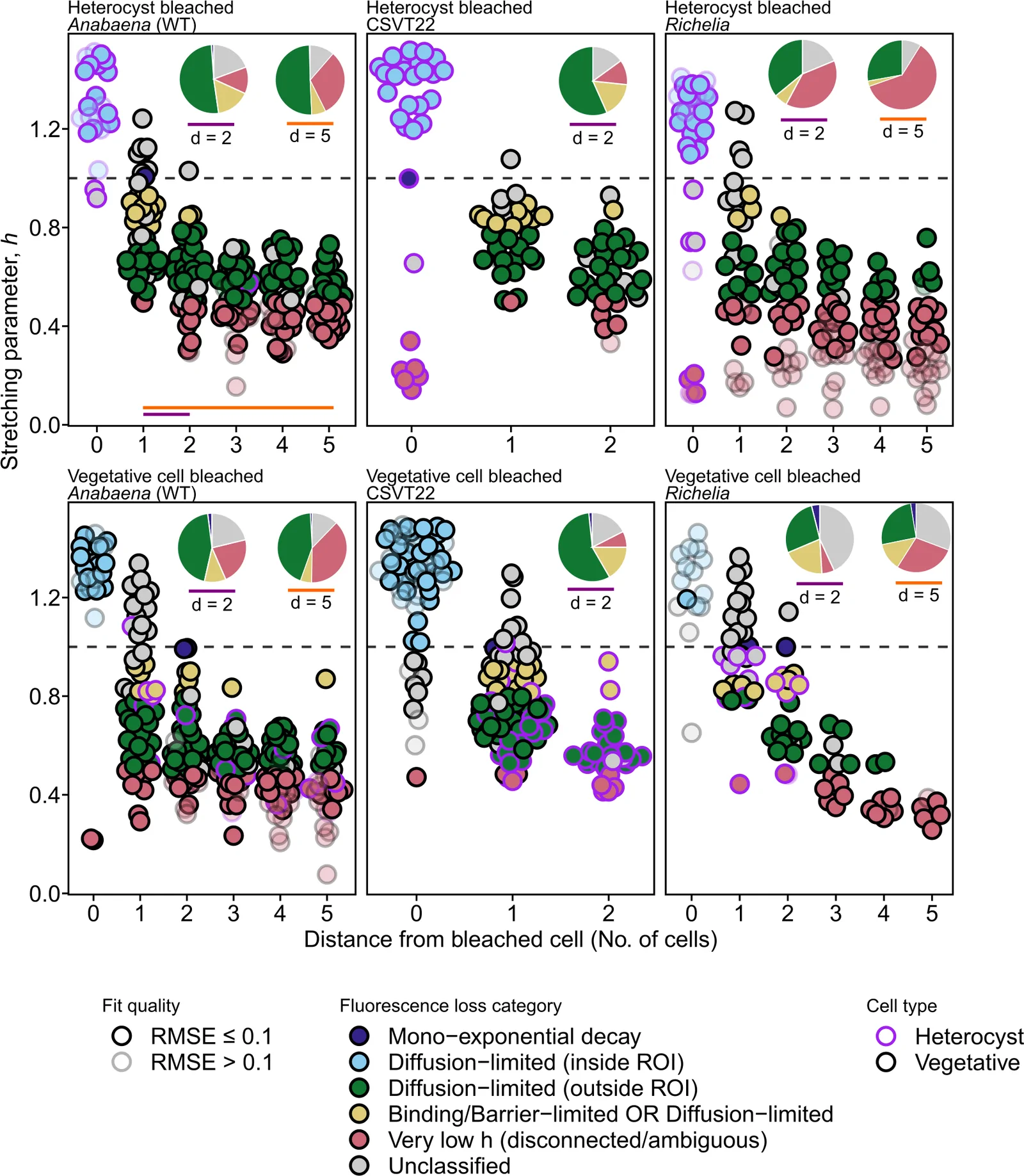
Physical classifications of single-cell fluorescence loss (FL) mechanisms. Scatterplots show the stretching parameter, *h*, as a function of distance from the bleached cell for individual cells, color-coded by FL category. The dashed line at *h* = 1 indicates mono-exponential behavior. Pie charts embedded in each facet panel summarize the proportional distribution of FL categories within the corresponding condition (species × bleached cell), in either within two-cell (*d* = 2, purple line) or five-cell (*d* = 5, orange line) distance from the bleached cell. Categories include mono-exponential decay (dark blue), diffusion-limited (inside ROI: light blue; outside ROI: green), binding/barrier-limited or diffusion-limited (yellow), very low *h* (red), and unclassified (gray). RMSE = StrExp model root mean squared error of residuals. Color for cell type indicates vegetative cells (black) and heterocysts (purple).

Finally, we examined the spatial distribution of fluorescence decay, which might reveal heterogeneity in intercellular exchange beyond that expected from simple diffusion. We focused on *Richelia* SC01 and *Anabaena* strains by comparing absolute differences in τ between cells on either side of the bleached cell. *Anabaena* showed consistently high asymmetry independent of cell type or neighboring cell configuration, suggesting substantial heterogeneity in intercellular exchange. In contrast, *Richelia* SC01 filaments with vegetative cells on both sides of the bleached cell showed significantly lower asymmetry, reflecting more balanced, symmetric propagation of fluorescence along the filament (Fig. 6). Together, these results indicate that cell type and filament organization are associated with distinct intercellular exchange patterns.

**Fig 6:**
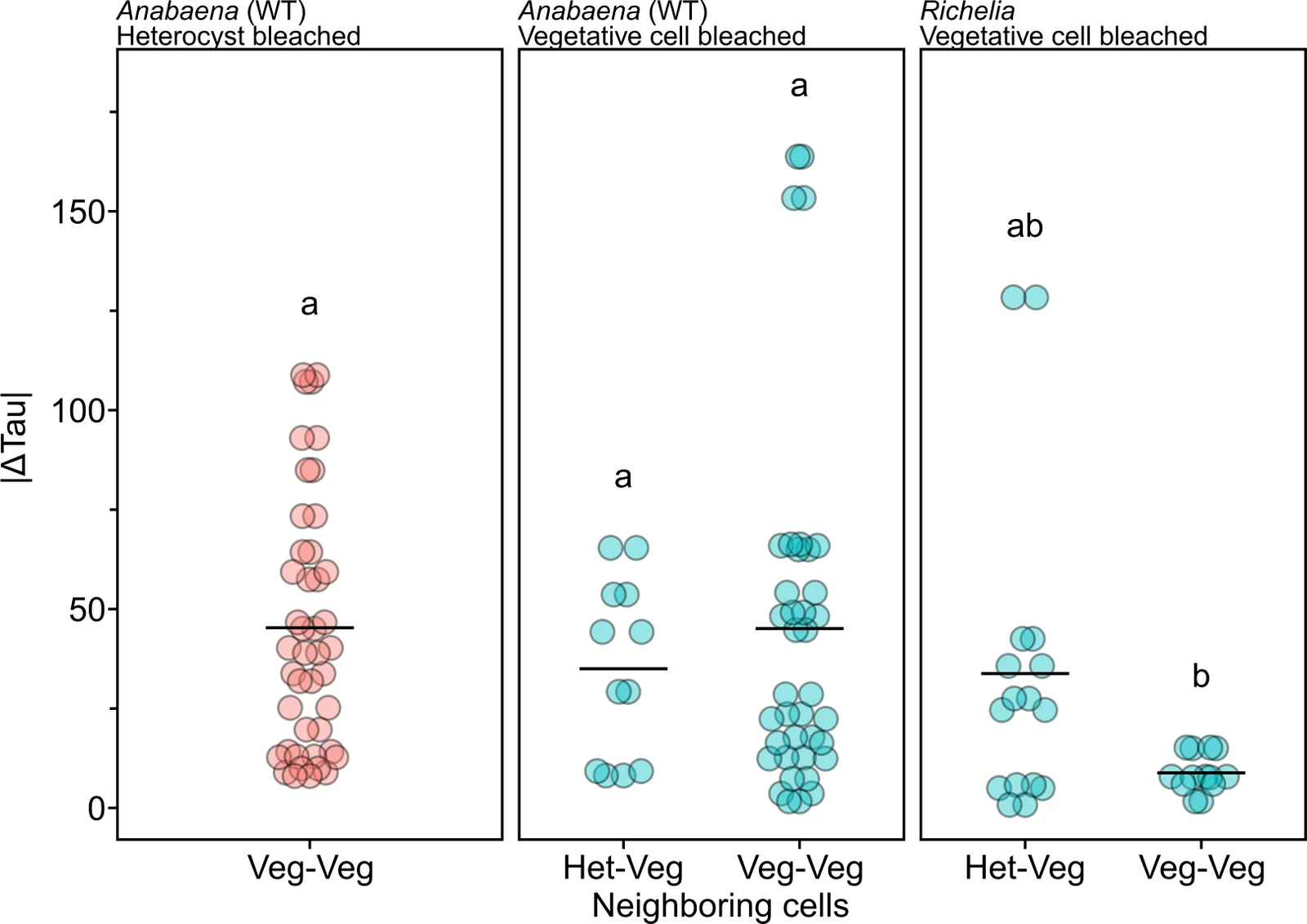
Asymmetry in intercellular exchange revealed by directionality scores. Directionality scores, defined as the absolute difference between recovery time constants (∣ΔTau∣) of left- and right-adjacent cell neighbors (Veg-Veg or Het-Veg) relative to the bleached cell, are shown for *Richelia* SC01 and *Anabaena* under different bleaching conditions (vegetative vs heterocyst). Each point represents a single filament, with mean values indicated by crossbars. Groups are significantly different if marked with different letters according to a Kruskal-Wallis/Dunn test (*H*[4] = 21.8, *P* < 0.001, *n* = 123 filaments). Higher directionality scores reflect greater asymmetry in intercellular communication across septa.

### Septal structure features of *Richelia* SC01 differ from *Anabaena* genotypes

To account for the selective change in intercellular communication, we isolated the peptidoglycan (PG; murein) sacculi from *Richelia* SC01 filaments and visualized them by TEM (Fig. 7; Fig. S5). In order to predict the cell type of the PG disks observed in the TEM micrographs, we compared the diameters of septal PG disks observed by TEM with the estimated diameters of heterocysts and vegetative cells measured in confocal laser scanning microscopy (CLSM) images. We also considered the likelihood of the cellular origin of the PG sacculi based on the heterocyst frequency (Fig. 1D and E). For example, the measurements by TEM and CLSM for vegetative cells of *Richelia* SC01 were similar (Fig. S6), and as such, the PG disks were likely derived from vegetative cells. The average septal PG disks were significantly larger in *Richelia* SC01 (2.77 ± 0.703 µm; *n* = 10) compared to reported measures of *Anabaena* vegetative cell septal PG disks (1.431 μm ± 0.413 µm; *n* = 22) and CSVT22 (0.952 μm ± 0.261 µm; *n* = 11) (44). Nanopore size was relatively consistent across septa and within the range reported for *Anabaena*, *Nostoc punctiforme*, and symbiotic *Nostoc azollae* (Table 4). In contrast, the number of nanopores per septal disk showed high variability, which may contribute to the observed variation in intercellular exchange patterns.

**Fig 7:**
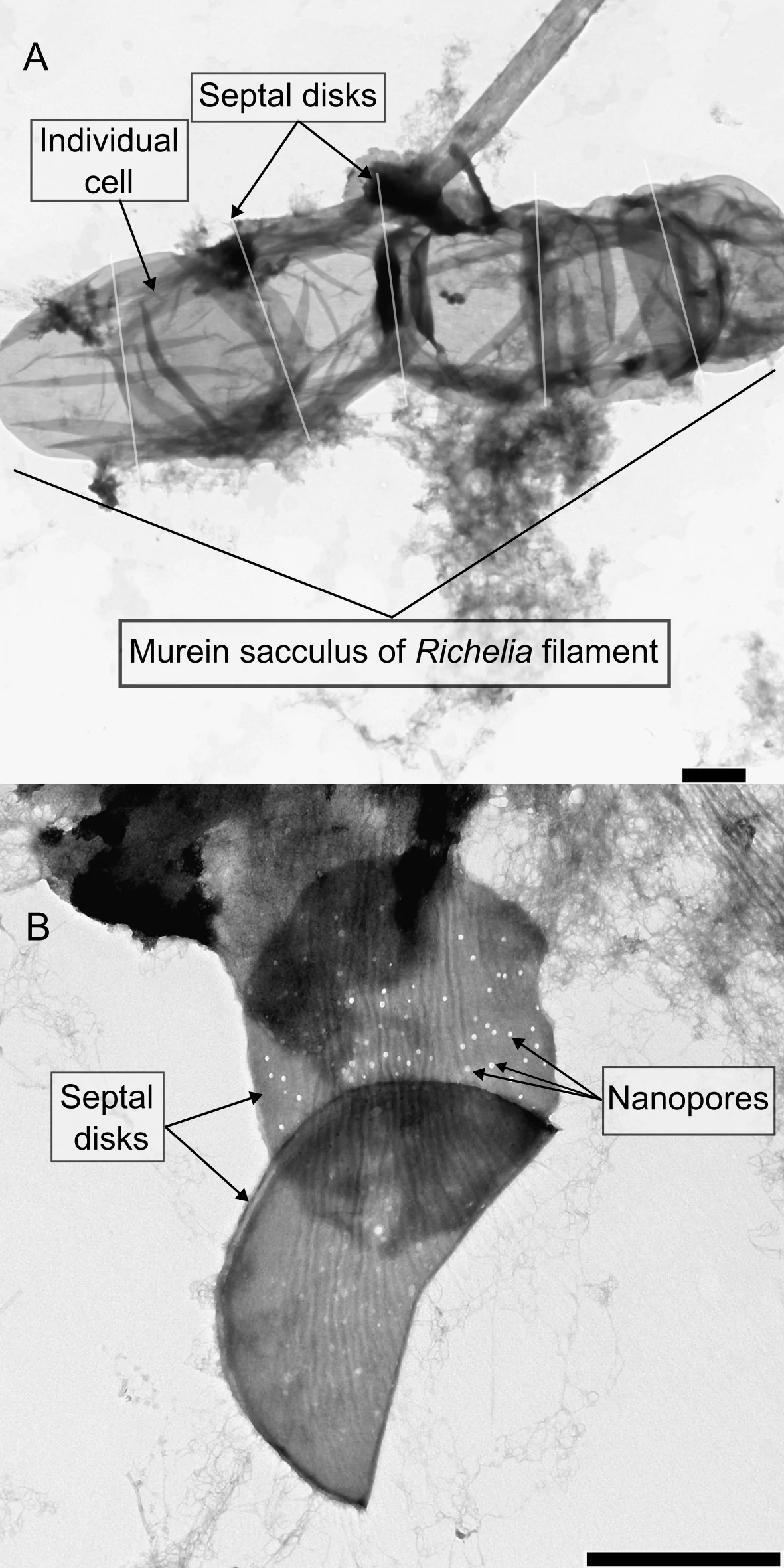
Example TEM micrographs of extracted peptidoglycan (murein) sacculi from *Richelia* SC01. (**A**) Murein sacculus of a six-cell filament of *Richelia* SC01. A few electron-dense regions are noticeable in the filament, some corresponding to the septal disks, while other regions likely result from the overlapping of PG layers from neighboring cells. (**B**) Examples of *Richelia* SC01 septal disks with visible nanopores. The bottom septal disk serves as an example of a fragmented septal disk, which was excluded from the analyses. Scale bars are 1 µm.

**TABLE 4:** Summary of nanopore parameters in various heterocyst-forming cyanobacteria^*a*^

Study	Strain	Lifestyle	Growth medium	Nanopores/disk, mean ± SD (*n*)	Nanopore diameter (nm), mean ± SD (*n*)
This study	*Richelia rhizosoleniae* SC01	FS	SO	76 ± 50.2 (10)	20.2 ± 9.4 (703)
44	*Anabaena* sp. PCC 7120	FL	BG11_0_	48.7 ± 12.3 (21)	20.3 ± 3.2 (383)
44	CSVT22 (*∆fraC-∆fraD*)	Mutant	BG11_0_C	7.3 ± 6.5 (11)	15.2 ± 5.0 (62)
45	*Anabaena* sp. PCC 7120	FL	BG11	50 ± 8 (5)	–
46	*Anabaena* sp. PCC 7120	FL	BG11_0_	48.75 ± 3.8 (5)	–
46	*Anabaena* sp. PCC 7120	FL	BG11	52.3 ± 6.2 (9)	–
47	*Anabaena* sp. PCC 7120	FL	BG11	–	19 ± 3.5 (524)
48	*Nostoc azollae*	OS	–	93 ± 83 (21)	19 ± 5 (306)
49	*Nostoc punctiforme*	FS	BG11	~50–150 (7)	–
17	*Nostoc punctiforme*	FS	BG11	155 ± 13 (8)	20 ± 1.8 (eight septal disks counted)

^*a*^ Measures mainly originate from vegetative cells. Allen and Arnon medium refers to the recipe described in Allen and Arnon (44). Only the BG11 medium contains added combined nitrogen unless stated otherwise. BG110 medium supplemented with 10 mM NaHCO_3_ is denoted by BG110C. SO medium is SN medium without added N as previously described (50) and made with a natural seawater base from the oligotrophic Sargasso Sea. – indicates that information was not specified in the study. FS, facultative symbiont; FL, free-living; OS, obligate symbiont; S, symbiotic.

Spearman correlation tests were run to identify relationships between septal disk area, nanopore abundance, and nanopore diameter of *Richelia* SC01. However, we found no significant correlations between any of the parameters (Fig. S7).

### Fra and AmiC homologs in the *Richelia* SC01 genome

To understand the possible molecular mechanisms enabling the single-cell selective control of intercellular communication we demonstrated above, we used sequences of *Anabaena* septal protein sequences as queries to identify putative homologs of FraC (WP_088243366.1, *E*-value = 4e^−40^), FraD (WP_088243365.1, *E*-value = 6e^−125^), FraE (WP_088243364.1, *E*-value = 4e^−111^), and SepJ (WP_088243279.1, *E*-value = 9e^−110^) in *Richelia* spp. strains (Table S1). Sequence alignments were performed in Clustal and confirmed that homologs were present in *Richelia* SC01 (Table S1). Based on the predicted synteny in the *Richelia* SC01 genome (RrhiSC01), *fraC*, *fraD*, and *fraE* form a gene cluster, while *fraE* was notably absent from the genomes of the other symbiotic *Richelia* spp. strains (RintRC01 and ReuHH01; Table S1). Using the nanopore-associated protein sequences of *Anabaena* as queries, putative homologs of AmiC1 (Alr0092) and AmiC2 (Alr0093) were identified in *Richelia* SC01 genome as (WP_088240876.1, *E*-value = 0.0), AmiC3 (All1140; WP_088240073.1, *E*-value = e^−158^), and the LytM regulatory factor Alr3353 (WP_088240267.1, *E*-value = e^−100^; Table S1).

## DISCUSSION

Filamentous cyanobacteria establish functional multicellularity through septal junctions that connect adjacent cells and permit gated exchange by simple diffusion (5, 29, 51), yet how cell type, filament architecture, and lifestyle shape exchange dynamics remains poorly understood. Here, we addressed this gap by comparing intercellular communication in three strains with contrasting filament architecture: *Anabaena* as an intercalary heterocyst-forming freshwater model; CSVT22 as a communication-impaired *Anabaena* mutant with terminal heterocysts (21); and *Richelia* SC01, a marine facultative diatom symbiont forming tapered filaments with terminal heterocysts (32, 52). Our study attempted to uncover how intercellular communication is controlled at both the single-cell and filament level.

Under nitrogen limitation, all strains resulted in the expected phenotype, including differentiated heterocysts, with morphological features (cell diameter and filament length) consistent with earlier reports (53). Successful 5-CF labeling was achieved in all three strains, including *Richelia* SC01 for the first time, demonstrating its suitability for studying intercellular communication in this cyanobacterium. It is important to note that fluorophores, e.g., 5-CF, calcein, and esculin, differ in molecular size (376.3, 622.5, and 340.3 Da, respectively), molecular charge, and immobility fractions, which alter the fluorophores’ effective exchange behavior (30). Therefore, observed differences between our results and previous studies may reflect tracer-specific properties, highlighting the importance of considering the fluorophore used in each study.

FRAP recovery rates in *Richelia* SC01 were similar to those of *Anabaena*, indicating that heterocyst position (terminal vs intercalary) does not strongly influence overall exchange capacity under the conditions tested. Here, *Richelia* SC01 and CSVT22 showed significant cell type-specific recovery rates, with vegetative cells recovering faster than heterocysts. Earlier FRAP studies (30, 42) have found similar results with *Anabaena* using different fluorophores (calcein and esculin, respectively). Combined, these results suggest that differential connectivity between cell types may represent a conserved feature among heterocyst-forming cyanobacteria rather than a strain-specific trait. Cyanophycin plugs at heterocyst cell poles may contribute to differential recovery between cell types by restricting inward fluorophore flow, as previously demonstrated in *Anabaena* mutants lacking polar cyanophycin (28, 40). Further supporting this, an *Anabaena* mutant with inactivated *fraH*, which produces heterocysts lacking the characteristic intracellular honeycomb membrane structures associated with cyanophycin plugs, showed increased rates of calcein transfer from vegetative cells to heterocysts compared to wild type (54). Notably, the *Richelia* SC01 genome encodes both cyanophycin synthase (WP_088243624.1) and cyanophycinase (WP_088243625.1), and cyanophycin granules have been observed in wild *Richelia* sp. (52). Hence, a similar impact of cyanophycin plugs acting as barriers to intercellular communication could explain a slower recovery in the heterocysts of *Richelia* SC01.

The presence of non-communicating cells observed here in both *Richelia* SC01 and *Anabaena* is consistent with previous observations in *Anabaena* (42), where communicating cells in *Richelia* SC01, together with the presence of putative homologs of FraC, FraD, FraE, and SepJ, suggest a similar regulatory mechanism potentially supporting coordinated intercellular exchange. Additionally, non-communicating heterocysts in *Richelia* SC01 may partly reflect senescent heterocysts, as previously noted for aging heterocysts in *Anabaena* filaments using esculin (30).

FLIP analysis complemented FRAP by allowing observations of the continuous fluorescence decay dynamics, and thus, we moved from a single-cell analysis to along filaments. Distance from the bleached cell was the strongest determinant of decay speed, consistent with diffusion-mediated exchange, but similar to the FRAP assays, strain- and cell type-specific differences emerged. Notably, CSVT22 showed the fastest fluorescence decay in FLIP despite displaying the slowest recovery in FRAP, and this pattern persisted even after accounting for the smaller filament size and correspondingly lower theoretical fluorescence reservoir of CSVT22 (Fig. 2E). These results in CSVT22 are consistent with previous evidence that loss of FraC/FraD function affects both septal connectivity and the regulation of intercellular exchange (20, 21, 55). The stretched exponential classification of decay profiles further supports our interpretation. In CSVT22, decay profiles rarely fell into ambiguous or disconnected categories, indicating that fluorophore exchange remained continuous across cells despite impaired regulation. In contrast, *Richelia* SC01 heterocysts frequently exhibited very low stretching parameter values, consistent with limited or heterogeneous connectivity.

Beyond differences in decay kinetics, FLIP revealed distinct exchange patterns between strains. *Anabaena* displayed consistently higher asymmetry in fluorescence loss patterns than *Richelia* SC01, independent of bleach cell type or neighboring cell identity. This asymmetry reflects differences in exchange between the two neighboring cells of a bleached cell rather than a directional bias of transport. The greater asymmetry observed in *Anabaena* may result from increased variability in septal junction activity among cells or from less coordinated regulation of septal junctions at opposite cell poles. Our data cannot distinguish between these possibilities, but they suggest that intercellular exchange is less uniformly organized in *Anabaena* than in *Richelia* SC01. Interestingly, despite its polarized filament morphology, including tapering and a terminal heterocyst, *Richelia* SC01 exhibited comparatively symmetric patterns among its vegetative cells. This suggests that morphological polarity does not necessarily translate into asymmetric intercellular exchange and points instead to a potential role for differences in the regulation of cell-cell communication. In the symbiotic context, where *Richelia* SC01 attaches to its diatom host at its terminal heterocyst (32), symmetric vegetative cell connectivity may facilitate efficient redistribution of fixed nitrogen from heterocysts toward vegetative cells and ultimately to the host, which is consistent with the rapid nitrogen transfer shown previously for this symbiosis (36). The reliance on a single terminal heterocyst may favor the maintenance of intercellular exchange along the filament, as disruption of connectivity could limit nitrogen transfer to cells located farther from the heterocyst. Furthermore, it is informative to consider heterocyst frequency in *Richelia* SC01 relative to other known cyanobacterial symbioses. Cyanobacteria, when associated with terrestrial plants, can exhibit substantial increases in heterocyst differentiation compared to when they are in a free-living state. Reported frequencies, however, vary widely and range 1%–60% depending on the symbiotic system (8). For example, frequencies of 10%–30% are reported in lichen symbioses compared to up to 60% in angiosperm-associated systems such as *Gunnera* (8). The heterocyst frequency of *Richelia* SC01 (7.9% ± 0.7%) is closer to values reported for free-living heterocystous cyanobacteria. Our demonstration of efficient and symmetric intercellular exchange in *Richelia* SC01 supports the capacity of this lineage to sustain such bidirectional metabolite redistribution along short filaments. A direct test of intercellular communication dynamics in the presence of the diatom host would be a valuable next step, as physical attachment and metabolic integration with the host may alter the asymmetry, magnitude, and rate of exchange observed here under free-living laboratory conditions.

To investigate whether the nanopore array could differ in *Richelia* SC01, the septal peptidoglycan of *Richelia* SC01 was extracted and visualized by TEM and compared with earlier reports of other cyanobacteria, including facultative (*N. punctiforme*) and obligate (*N. azollae*) symbionts of plants. This is consistent with the presence of orthologs of key *Anabaena* proteins (AmiC1: Alr0992; AmiC2: Alr0093; AmiC3: All1140; and the AmiC regulatory factor LytM: Alr3353) encoded in the *Richelia* SC01 genome involved in nanopore formation in septal disks (56). The nanopore size and abundance in *Richelia* SC01 were within the range reported for other heterocystous cyanobacteria, including both free-living and symbiotic strains. Although *Richelia* SC01 vegetative cells possessed significantly larger septal disks than those of *Anabaena*, nanopore abundance was similar between strains, resulting in a lower nanopore abundance per unit disk area in *Richelia* SC01. The septal disk area, nanopore abundance, and nanopore diameter of *Richelia* SC01 were, however, not significantly correlated (Fig. S7), indicating that these structural features vary independently. Additionally, there was high variation in nanopores per disk, and occasionally larger pores were observed in *Richelia* SC01 septa, which is similar to observations recently reported in septal disks of vegetative cells and a hormogonia mutant (∆*fraI*) of the facultative symbiont *Nostoc punctiforme* (49). As suggested by Janović et al. (49), the presence of larger pores may explain higher rates of communication in some cells.

More broadly, our findings contribute to a growing appreciation that regulated intercellular communication through cytoplasmic bridges is a recurrent and functionally important feature across the tree of life. Chaigne and Brunet (6) proposed that cytoplasmic bridges may have first served a structural role in the evolution of multicellularity before later enabling nutrient exchange and intercellular communication. Previous studies have established that septal junction structure and regulation govern intercellular communication. Our results further show that cell identity and filament organization influence how exchange is distributed within multicellular filaments, indicating that cyanobacterial multicellularity is shaped by the integrated effects of structural connectivity, regulatory control, and filament architecture.

## MATERIALS AND METHODS

### Strains and growth conditions

*Anabaena* sp. strain PCC 7120 (7) and the *Anabaena* ∆*fraC-*∆*fraD* double mutant (CSVT22) (21) were grown photoautotrophically in liquid BG11 medium (7) under continuous shaking (100 rpm) and constant light (42 μE m^−2^ s^−1^) at 30°C. The ferric ammonium citrate in the original medium recipe was replaced with ferric citrate (Sigma-Aldrich). Heterocyst differentiation was induced in *Anabaena* and CSVT22 by harvesting filaments grown on BG11 (5–7 days after inoculation), washing three times with BG11_0_ medium (lacking NaNO_3_), and resuspended in the same medium for 48 h prior to live-cell imaging experiments. *Richelia* SC01 was grown photoautotrophically in SO medium (SN lacking NaNO_3_) (57) on a 14 h:10 h light:dark cycle (15 ± 5 μE m^−2^ s^−1^) at 26°C under continuous shaking (80 rpm, 10 mm radius of rotation). *Richelia* SC01 was inoculated into fresh SO medium 6–7 days prior to live-cell imaging experiments.

### Filament morphology

Triplicate cultures of each strain were grown under their respective conditions (BG11_0_ for 48 h for *Anabaena* and CSVT22, and SO medium for *Richelia* SC01), and brightfield micrographs were acquired to quantify filament length, heterocyst frequency, and heterocyst position. For each replicate, 100 filaments were measured to capture intra-strain variability, and only intact filaments were included in the analysis. Heterocysts of *Anabaena* and CSVT22 were identified by mixing cell solutions (1:10) with alcian blue (1% [wt/vol] in 95% ethanol) for 5–10 min (58). See the supplemental methods for details on counting criteria (Table S4). Images of wild *Richelia rhizosoleniae* were obtained from an image archive of plankton from the *Tara Oceans* (37) under accession number S-BSST529 at the EMBL-EBI repository BioStudies (http://www.ebi.ac.uk/biostudies).

### Purification, imaging, and analysis of murein sacculi from *Richelia* SC01

*Richelia* SC01 cells in late stationary growth phase (20–30 days after transfer to SO media) were harvested by gravity filtration (Whatman Cyclopore Track Etched Membrane, 47 mm, 5 µm pore size), flash frozen in liquid nitrogen, and stored at −80°C until further handling (hereafter, pellet). The pellets were processed as previously described (17). Samples were then imaged with a Zeiss Libra 120 Plus electron microscope at 120 kV. See the supplemental methods for details on processing of the TEM micrographs.

### FRAP and FLIP analyses

Aliquots (1 mL) of cell culture were harvested by centrifugation, washed in respective N-deplete growth medium (BG11_0_ for *Anabaena* and CSVT22 and SO for *Richelia* SC01), resuspended in 1 mL of fresh growth medium, and amended with 4 µL 5-carboxyfluorescein Diacetate, Acetoxymethyl Ester (5-CF; Molecular Probes, Invitrogen) in Dimethyl sulfoxide (5 mg/mL). Initial tests with calcein (Molecular Probes, Invitrogen) in *Richelia* SC01 showed no uptake of the dye; therefore, we used 5-CF (data not shown). All centrifugation steps here and after were at a speed of 20,800*g* and 1 min in duration. Subsequently, the cell suspension was incubated in the dark in respective growth conditions (see above) for 1 h. Filaments were then processed as previously described (21). Small agar blocks were cut out and transferred to 35 mm µ-dishes (Ibidi GmbH, Graefelfing, Germany), and measurements were carried out at respective growth temperatures, i.e., 30°C for *Anabaena* and the strain CSVT22 and 26°C for *Richelia* SC01. Note that experiments with *Anabaena* and CSVT22 were carried out within 48 h of inducing heterocyst differentiation by removal of N. For strain CSVT22, only filaments with two vegetative cells and a terminal heterocyst were chosen for the experiments.

FRAP and FLIP experiments were carried out with a Zeiss LSM 780 confocal laser scanning microscope and a Plan-Apochromat 63×/1.4 Oil DIC M27 objective (Zeiss, Germany). The samples were excited with the Argon laser (488 nm), and emission of 5-CF was observed at 500–527 nm with the use of the main dichroic beam-splitter 488. Uptake of 5-CF in *Richelia* SC01 could be observed and differentiated from autofluorescence (Fig. S8). Images (512 by 512 pixels and 16-bit depth) were acquired with laser power set to 0.3%–2.5%, and care was taken not to saturate the detector (GaAsP) by adjusting the gain between 500 and 700. The pinhole was set to 150 µm for all experiments. Only filaments that fit within the field of view were observed and measured. For most images, a zoom of 1.8× was used, except for longer *Anabaena* filaments that did not fit the frame, for which a zoom of 1.2×–1.5× was used. Parallel brightfield images were acquired for *Richelia* SC01. The frame interval for both FRAP and FLIP experiments was 1 s, and three prebleach images were acquired prior to the start of an experiment. For FRAP experiments that lasted 30 s, the recovery of fluorescence was measured for 27 frames after one photobleaching event (100% laser power, 30 iterations). For FLIP experiments that lasted 60 s, photobleaching occurred at 10 s intervals (80% laser power, 30 iterations), and the decay of fluorescence in every cell within a filament was measured between bleaching events (six bleaching events in total).

### Data analysis and visualization

Data analyses and visualization were done in R (v. 4.3.1) using the “tidyverse” (2.0.0) package collection (59). Kruskal-Wallis/Dunn tests were performed using the “tukeygrps” package (https://github.com/leonardblaschek/tukeygrps). Note that the statistical methods differ from previous studies where Mann-Whitney *U* tests were used. Chi-squared/pairwise proportion tests were performed using the “rstatix” package (60). All statistical tests were done with ɑ = 0.05. See Table S2 for a detailed list of R packages and versions used. All image analyses were done in Fiji (61). Statistical results are provided in Table S3.

During FRAP analyses, the average intensities for every time point were recorded for each bleached cell. In case there was significant movement of filaments, image stacks were aligned prior to further analysis. FRAP models were fitted to the data by using the “nls.multstart” package (62). FRAP data were processed by background subtraction and then range normalization using the following formula:


(1) $$I=\frac{I(t)-I_{\min}}{I_{\max}-I_{\min}},$$

where *I*(*t*) is the fluorescence intensity in the bleached cell at time *t*, and *I*_min_ and *I*_max_ are the minimum (directly after photobleaching) and maximum (prebleach) intensities, respectively. Recovery rates were quantified using previously reported FRAP models (28, 45). The mono-exponential models were fitted depending on whether the bleached cell was connected to one,


(2) $$C_{B}=C_{0}+C_{R}\left(1-e^{-Rt}\right),$$

or two cells,


(3) $$C_{B}=C_{0}+C_{R}\left(1-e^{-2Rt}\right),$$

where *C_B_* is the fluorescence in the bleached cell, which tends toward (*C_0_ + C_R_*), *C_0_* is the fluorescence immediately after the photobleaching, *t* is the time, and *R* is the recovery rate. Cells for which the FRAP models could not be fitted were excluded from the data set, and cells with non-significant fits (*P* ≥ 0.05) were assigned *R* = 0 s⁻¹ and classified as non-communicating (see the supplemental methods).

FLIP image time-series were loaded into Fiji, then processed and analyzed with the PixBleach plugin, which fits exponential decay models (non-linear regression) on a pixel-by-pixel basis (43, 63). The images were first pre-processed by (i) smoothing with a Gaussian filter (standard deviation = 0.5) in the spatial and temporal dimensions, and (ii) only pixels that had a minimal decay of 100 were selected. Then, a stretched exponential (StrExp) model was fitted to the processed images (Fig. S9):


(4) $$I(\vec{r},t)=I_{0}(\vec{r})\times \exp\left[-{\left(\frac{t}{\tau (\vec{r})}\right)}^{h(\vec{r})}\right]+I_{\infty }(\vec{r}).$$

Here, the fluorescence loss at each pixel position $\vec{r}=(x,y)$ due to repeated bleaching in the ROI is defined as $I\left(\vec{r},t\right)$. $I_{0}$ is the pre-bleach intensity, τ is the decay time constant (in seconds), and $I_{\infty }$ is the intensity at infinite time and often approximates the background noise level. To assess the quality of the StrExp model fits, we calculated the RMSE of residuals for each cell (Fig. S10A; Fig. S11A). RMSE varied with both distance from the bleached cell, bleached cell type, and strain (Fig. S10B and C). The RMSE was included as a fixed effect in subsequent statistical models (linear mixed models) to account for parameter uncertainty. The model has a stretching parameter (*h*), which describes the stretching or the compression of the decay, and a time constant (τ) describing the speed of the decay (Fig. S11B and C). The decay in the StrExp function is described by:


(5) $$k(t)=\frac{1}{h\tau_{0}}\times {\left(\frac{t}{\tau_{0}}\right)}^{\frac{1}{h}-1}.$$

The *h* switches from *h* > 1 (stretching) for distances closer to the bleached ROI and the depletion zone to *h* < 1 for distances further away from the bleach spot. When *h* = 1, the decay is described as mono-exponential, meaning *k*(*t*) ≈ 1/τ_0_. τ_0_ is the characteristic decay time constant from the StrExp fit (Fig. S11D).

All data were background-corrected and min-max normalized (scaled from 0 to 1 per cell; equation 1) to ensure that our model parameters were independent of absolute intensity variation (Fig. S12) and reflected only the relative decay dynamics (Fig. S13). *Richelia rhizosoleniae* SC01 filaments, which had two heterocysts per filament (two filaments in total), were excluded from figures and analyses.

## Data Availability

Original TEM micrographs (from this study) and FRAP/FLIP micrographs can be made available upon request. Data for all figures, FRAP/FLIP image metadata, and FRAP model parameter results can be accessed through https://doi.org/10.6084/m9.figshare.33208233.

## SUPPLEMENTAL MATERIAL

Supplemental figures aem.00935-26-s0001.pdf Figures S1 to S14 and Table S1.

Supplemental methods aem.00935-26-s0002.docx Supplemental methods.

Table S2 aem.00935-26-s0003.xlsx Summary list of R packages used in this study.

Table S3 aem.00935-26-s0004.xlsx Summary of statistical analyses.

ASM does not own the copyrights to Supplemental Material that may be linked to, or accessed through, an article. The authors have granted ASM a non-exclusive, world-wide license to publish the Supplemental Material files. Please contact the corresponding author directly for reuse.
