# Supplementary material for: Cell identity and filament architecture shape intercellular communication in the terminal heterocystous cyanobacterium Richelia rhizosoleniae SC01
Source: Appl Environ Microbiol. 2026 Sep 10;92(10):e00935-26. doi: 10.1128/aem.00935-26 (PMC13689106; doi:10.1128/aem.00935-26)
Supplement: Supplementary file 1 [file aem.00935-26-s0001.pdf]

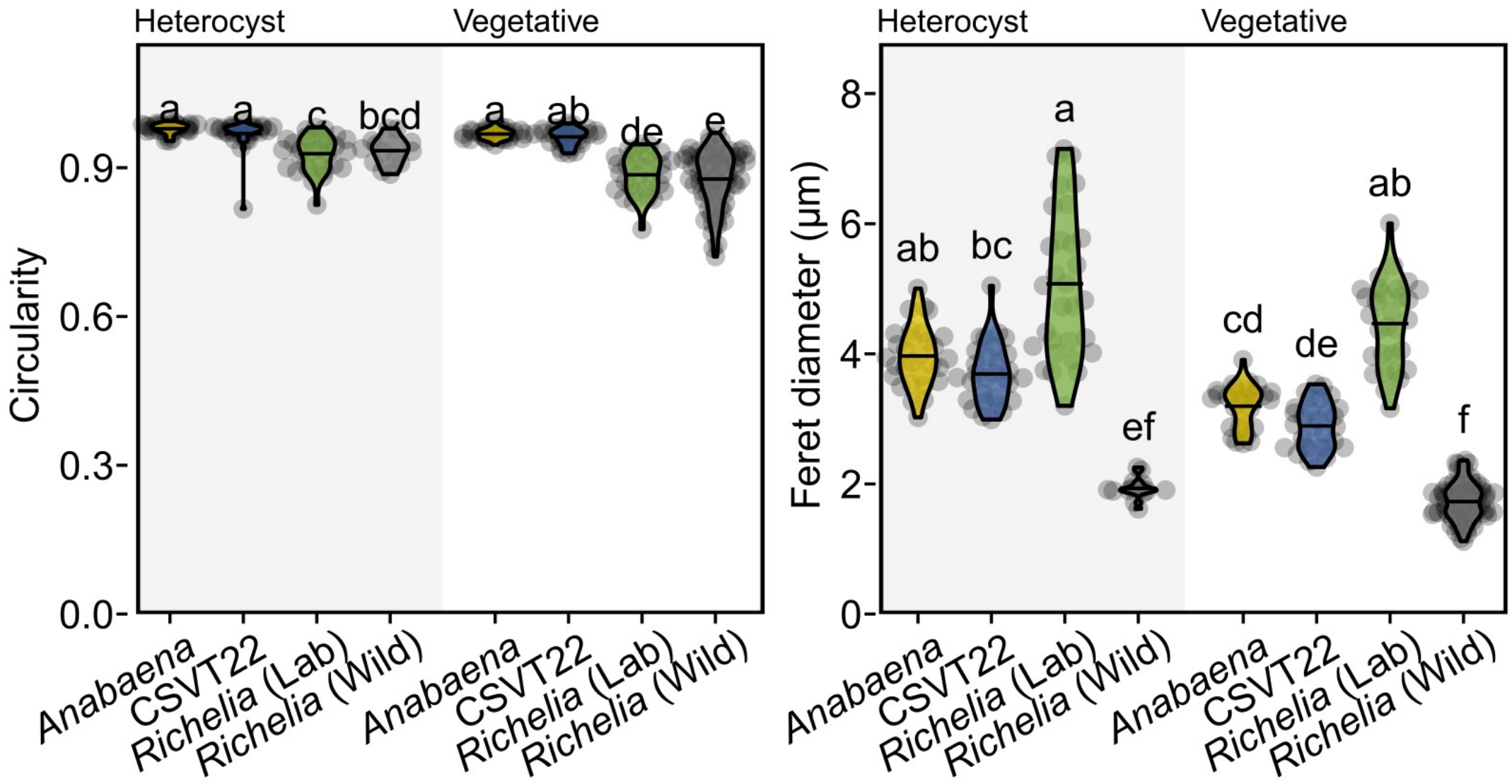

Figure S1 | Cell morphology characteristics of *Anabaena* sp. PCC 7120 (Wildtype and ∆*fraC*-∆*fraD* mutant) and *Richelia rhizosoleniae* (lab strain SC01 and wild).
(**A**) Cell circularity measurements for each cyanobacterial strain. The circularity ranges from 0 (most elongated) to 1 (perfect circle). Groups are significantly different if marked by different letters according to a Kruskal-Wallis/Dunn test ($H[7] = 178.9$, $p < 0.001$, $n = 263$ cells). (**B**) Cell diameter (Feret) measurements for each cyanobacterial strain. Groups are significantly different if marked by different letters according to a Kruskal-Wallis/Dunn test ($H[7] = 225.8$, $p < 0.001$, $n = 263$ cells).

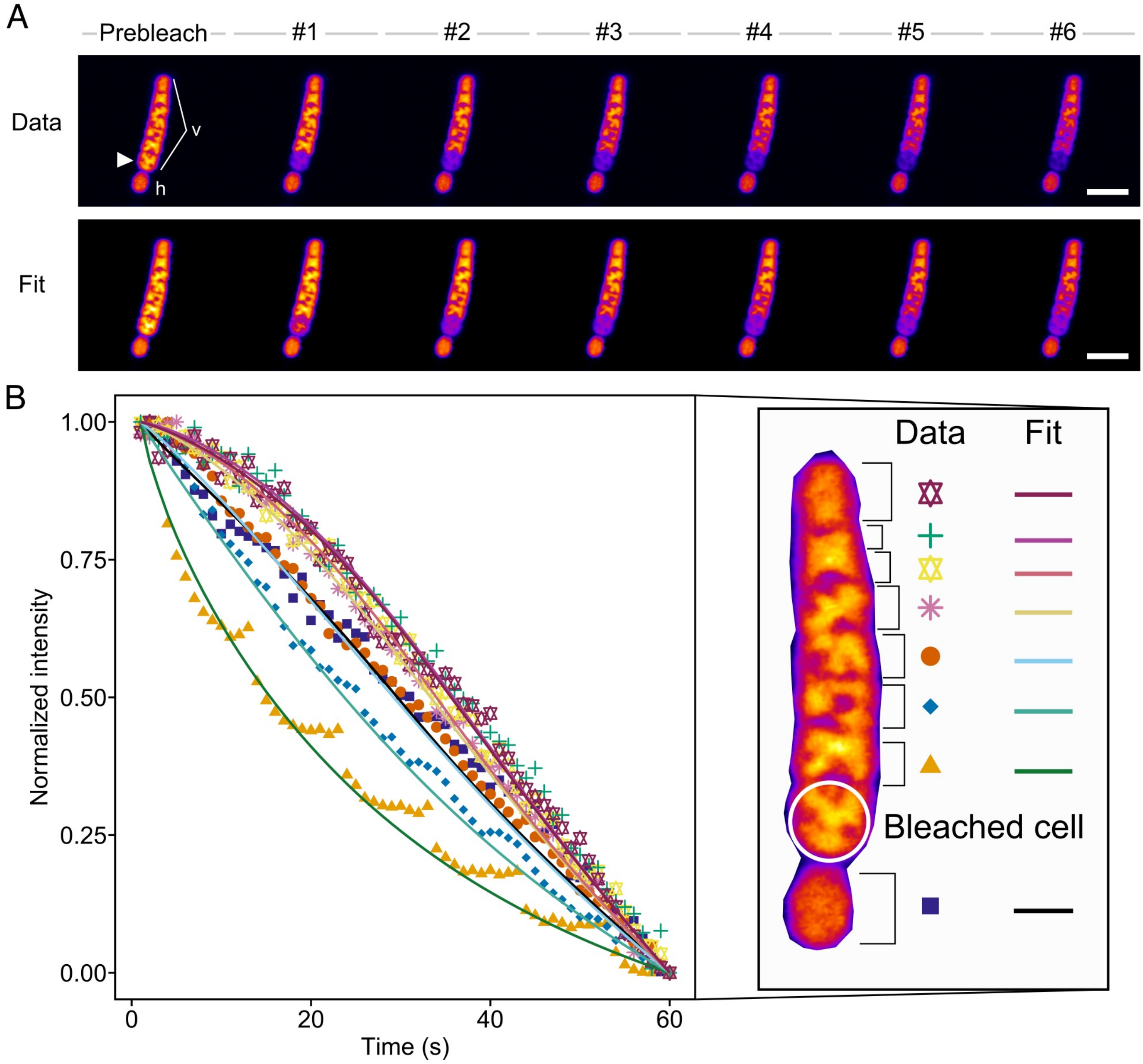

Figure S2 | Results from *Richelia rhizosoleniae* SC01 FLIP experiment and analysis.
(**A**) FLIP experiment example in which a vegetative cell of Richelia was photobleached (white arrow). A montage of each photobleaching event (#1-6, 10 s intervals) of original data (upper panel, 'Data') or of the reconstruction from the pixel-wise fitting of the StrExp function (lower panel, 'Fit'; see Eq. 4). The scale bar is 10 μm. (v, vegetative cells; h, terminal heterocysts).
(**B**) Normalized intensity for original data (points) and StrExp function fits (lines) for each of the corresponding cells in the filament from panel A (see legend to the right).

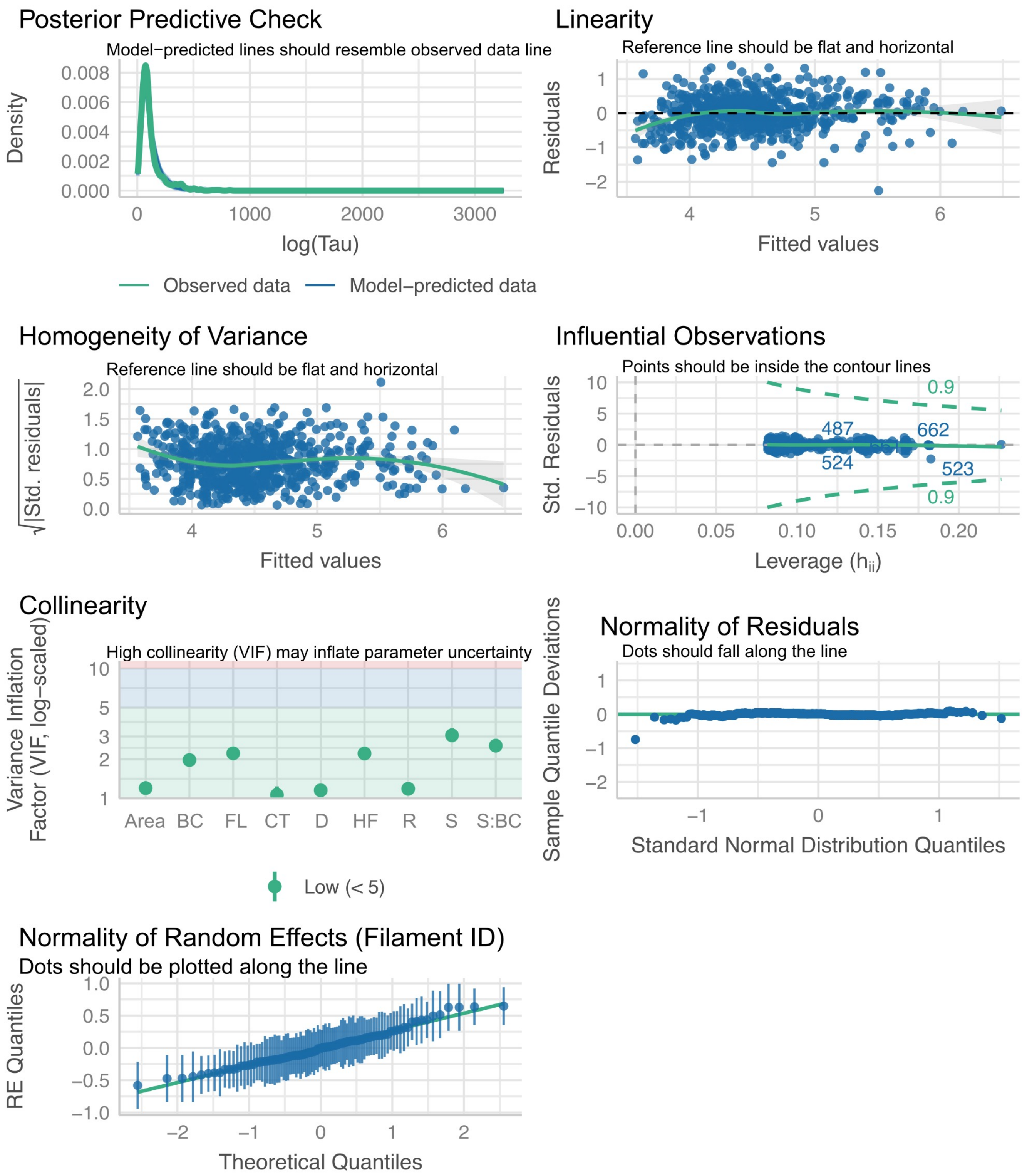

Figure S3 | Model diagnostics for linear mixed-effects analysis of the FLIP time constant (Tau). Diagnostic plots for the linear mixed-effects model fitted to log-transformed FLIP time constants (Tau). The model included fixed effects of distance from the bleached cell (D), strain (S), bleached cell type (BC), heterocyst frequency (HF), total number of cells per filament (FL), cell type (CT), cell area (Area), and residual error/RMSE (R), with random intercepts for sample identity (Filament ID). Plots show residual distributions, variance homogeneity, collinearity, normality of residuals, and influential observations. The absence of strong deviations supports that model assumptions were adequately met.

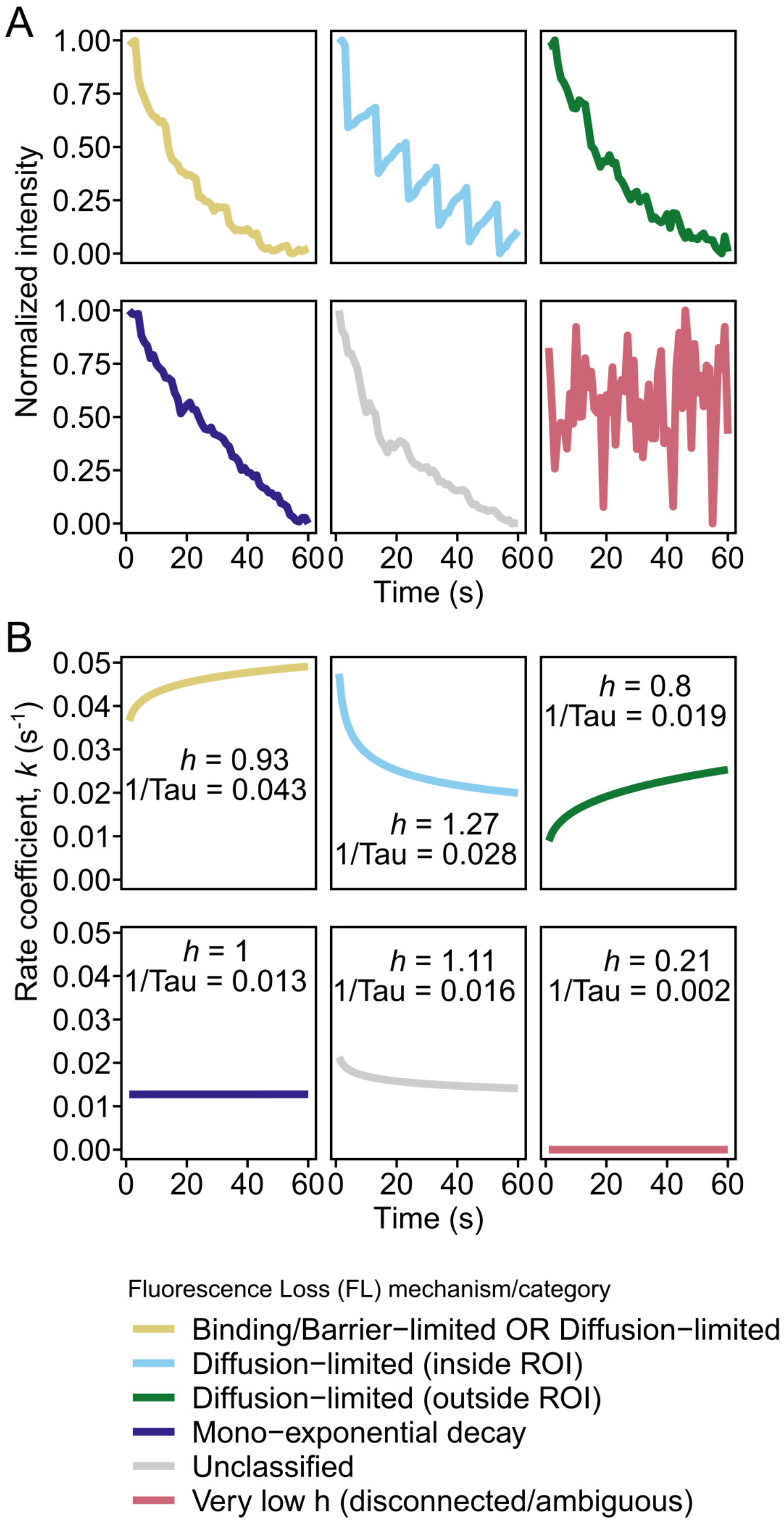

Figure S4 | Example cells from *Richelia* filaments were randomly selected within 5 cells of the bleached cell to represent typical decay behaviors for each fluorescence loss (FL) category.

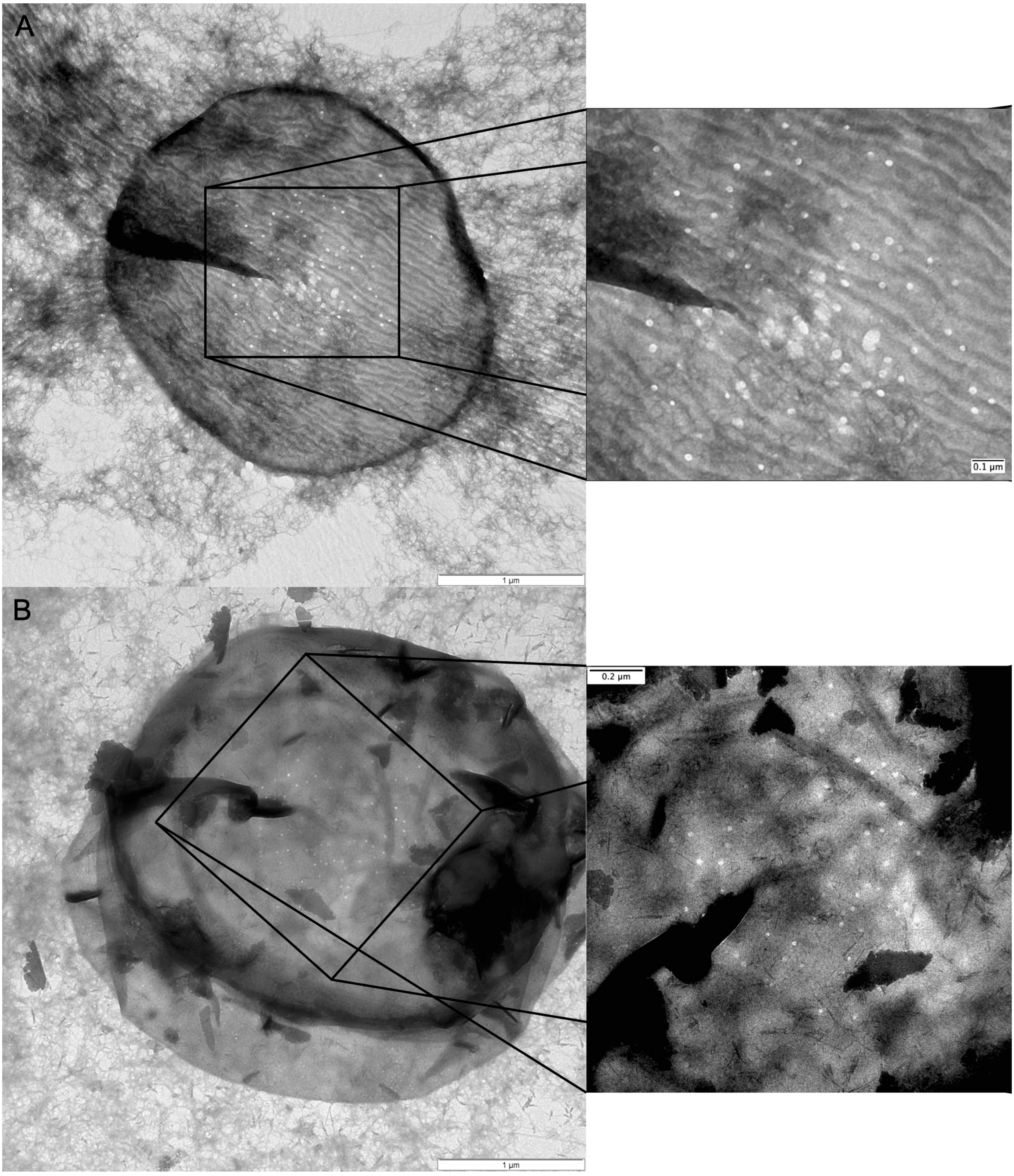

Figure S5 | A-B Transmission electron microscopy (TEM) micrographs of septal peptidoglycan disks of *Richelia* SC01 with nanopore arrays. Images to the right are zoomed in micrographs of the black squares in the micrographs to the left.

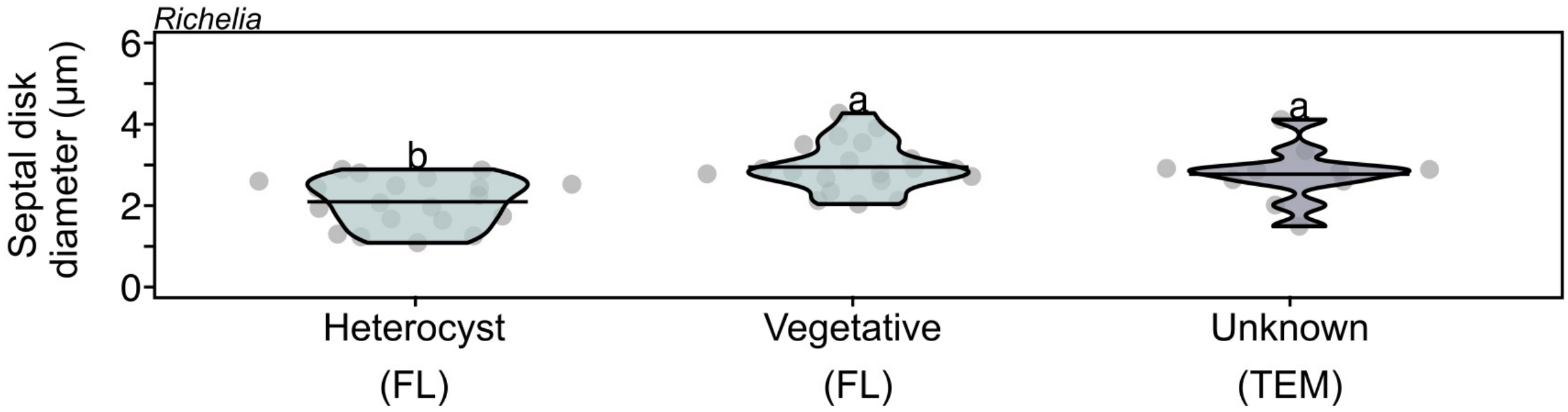

Figure S6 | Pairwise comparisons between septal diameters of randomly selected cells of *Richelia rhizosoleniae* SC01 from confocal laser scanning microscopy images (FL; n(Heterocyst) = 20, n(Vegetative) = 20) and septal peptidoglycan disk diameters (TEM; n = 10). The central lines indicate the median. Groups marked with different letters are significantly different according to three Kruskal-Wallis/Dunn tests (H[2] =, $p < 0.001$, n = 50 septal disks).

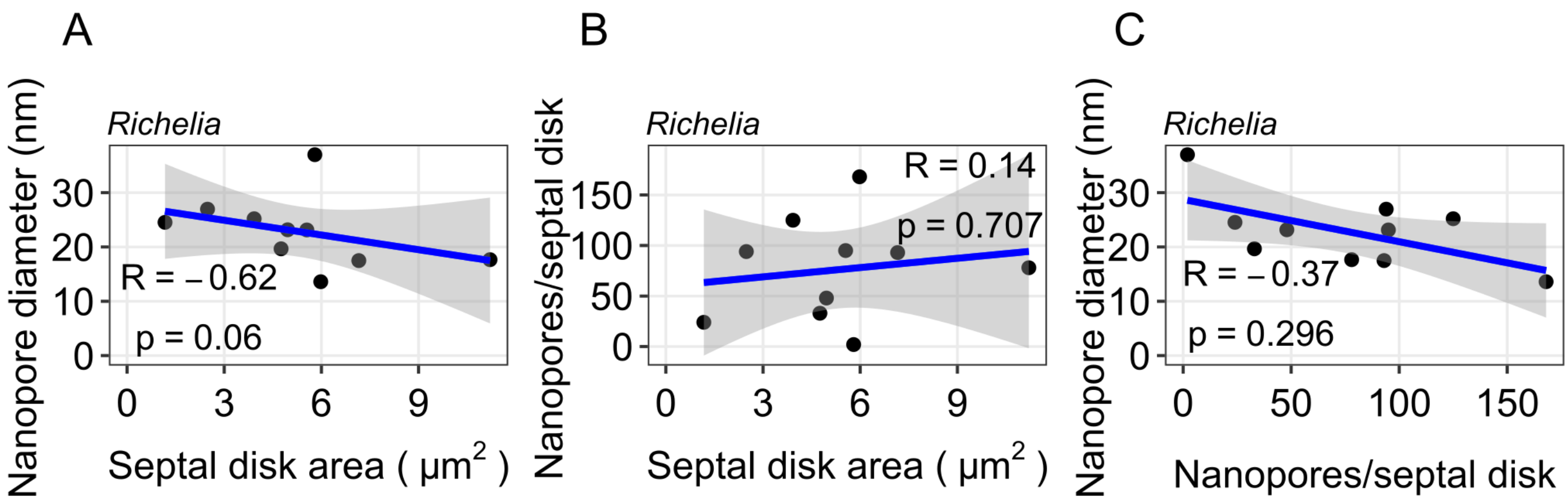

Figure S7 | Correlation tests (Spearman) between different nanopore and septal peptidoglycan disk characteristics in *Richelia* SC01. (**A**) Correlation between nanopore diameter and septal disk area. (**B**) Correlation between nanopores/septal disk and septal disk area. (**C**) Linear regression between nanopore diameter and nanopores/septal disk. An α = 0.05 was used for all linear regressions.

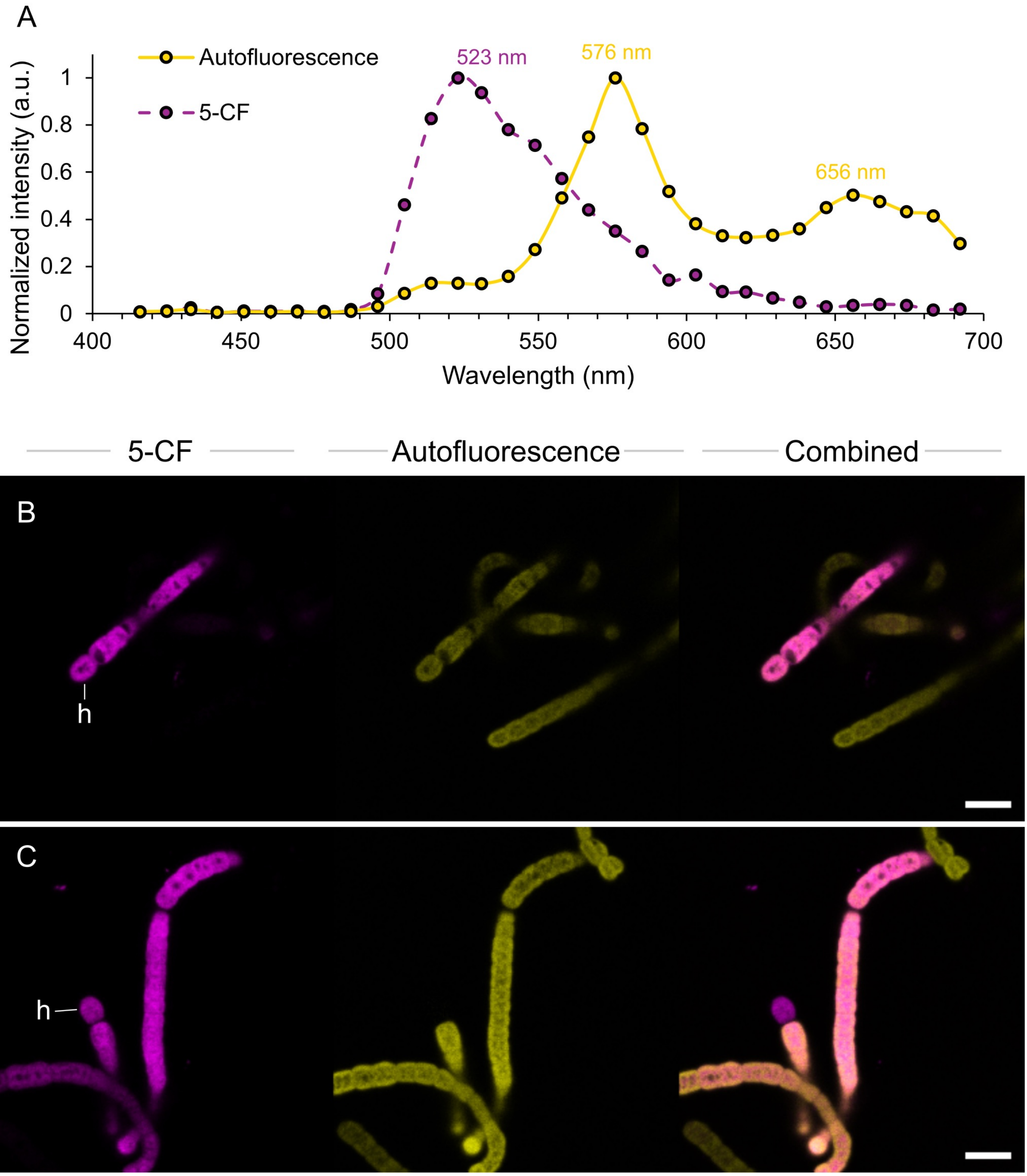

Figure S8 | 5-CF labelling in *Richelia* SC01. (**A**) Spectra of *Richelia* SC01 autofluorescence and 5-CF. Emission maxima are indicated above each peak with corresponding color code. (**B-C**) Examples of 5-CF labelling in *Richelia* SC01 filaments. *h* indicates heterocyst and the rest of the cells are vegetative cells. Scale bars are 10 µm. The images were derived from lambda scans with 8.9 nm sections in the λ-dimension (32-channels) and the fluorescent species were separated using linear unmixing in FIJI/ImageJ.

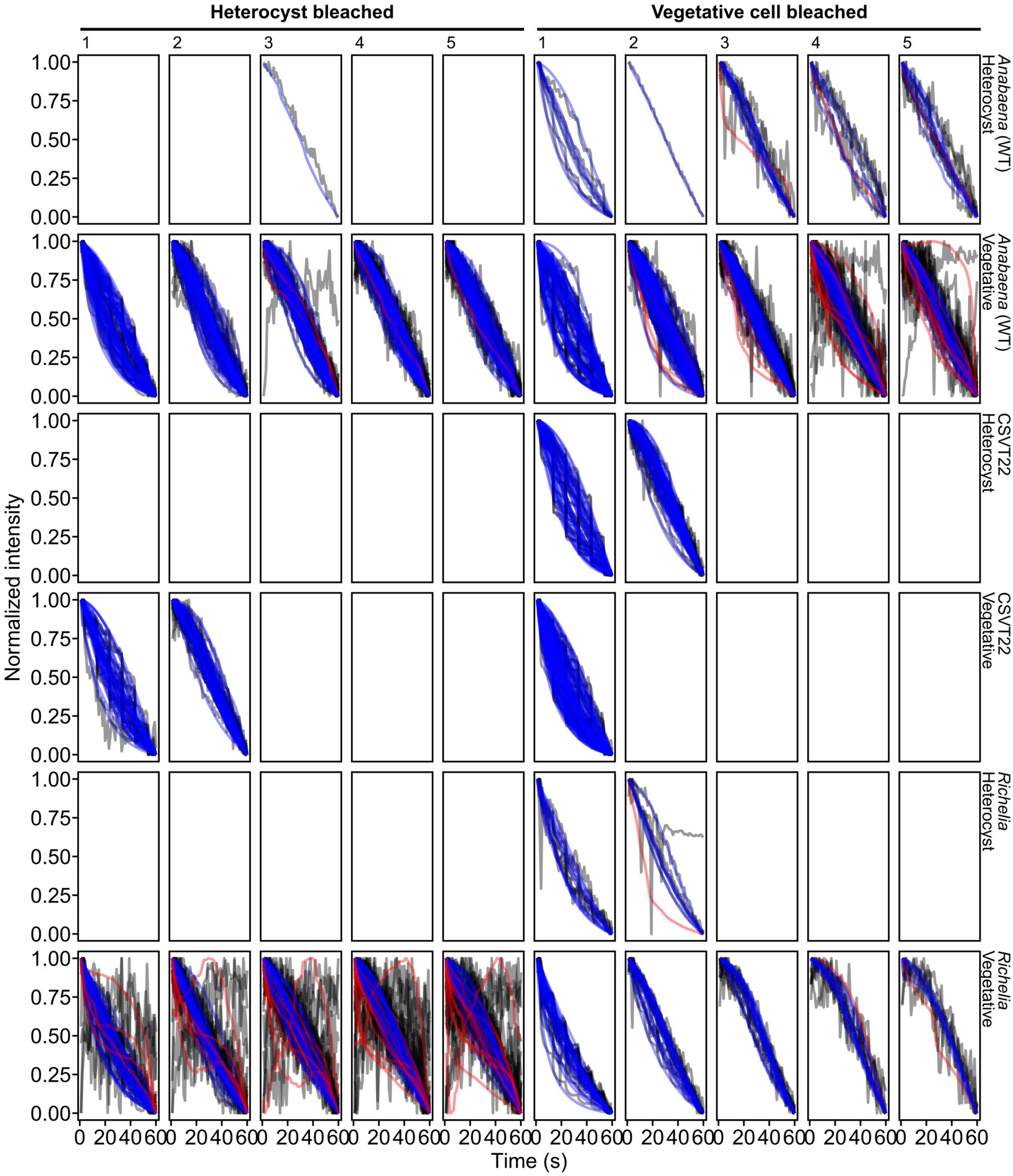

Figure S9 | Normalized fluorescence decay curves for the FLIP data (cells that are between 1-5 cells away from bleached cell; black lines) and corresponding fitted StrExp functions (red or blue lines) for the strains *Anabaena* sp. PCC7120, *Anabaena* Δ*fraC*-Δ*fraD* double mutant (CSVT22) and *Richelia* SC01. RMSE = Root Mean Squared Error. Red lines indicate RMSE > 0.1 (bad model fit) while blue lines indicate RMSE ≤ 0.1 (good model fit). Panel rows indicate the strain and cell type while columns indicate the bleached cell type and distance from bleached cell in number of cells.

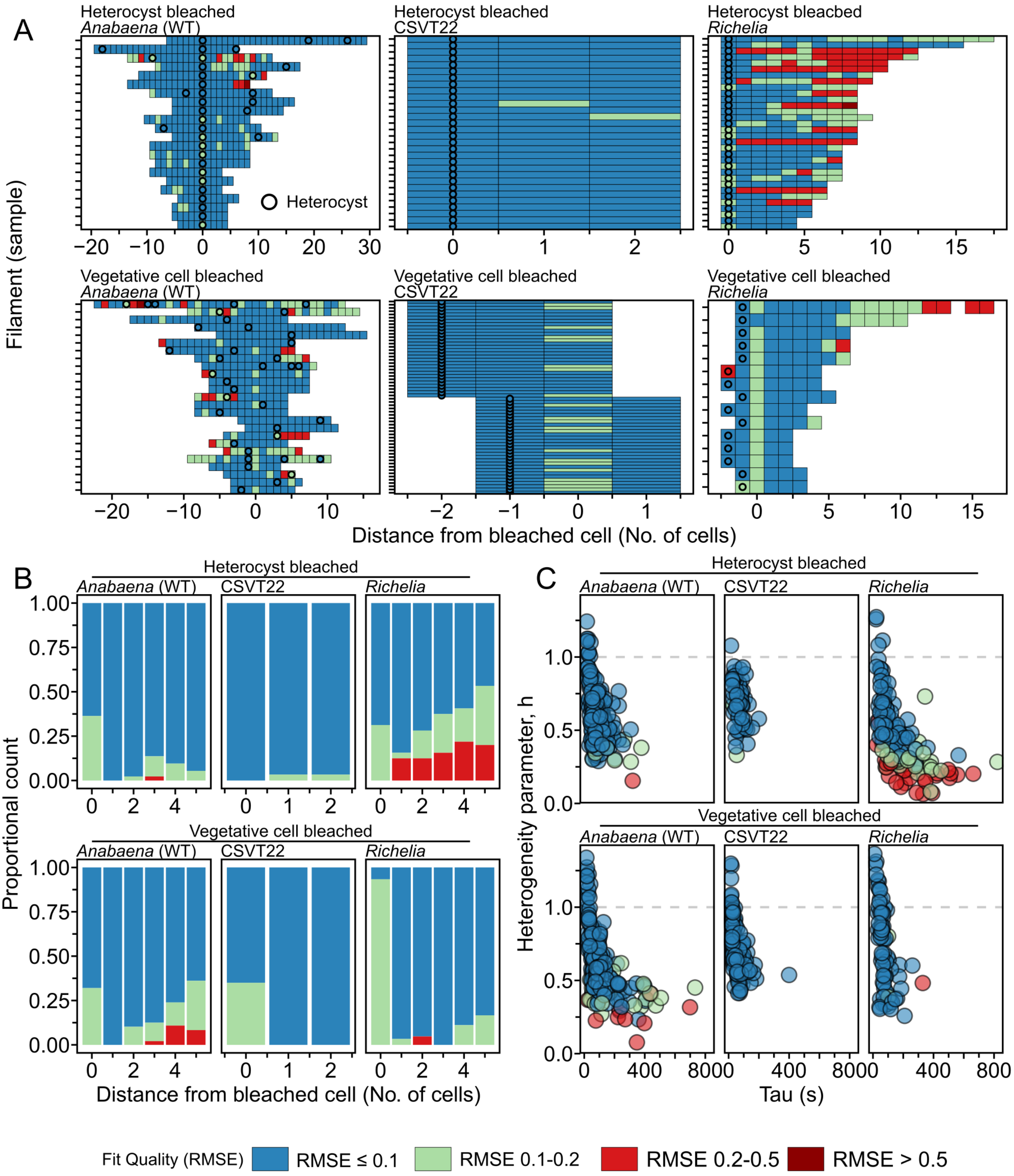

Figure S10 | RMSE was computed for each cell as the square root of the mean squared difference between the observed and fitted normalized intensity decay curves. It serves as a goodness-of-fit metric for the stretched exponential model used in FLIP analysis. Low RMSE values indicate reliable model fits and interpretable decay kinetics, while high RMSE values flag noisy or ambiguous dynamics. (A) All cells in every filament (rows) colored according to an RMSE range. Cells within a 5-cell distance from the bleached cell were filtered for further analyses, including proportions of each category for each distance (B) and distributions of model parameter estimations of *h* and Tau (C).

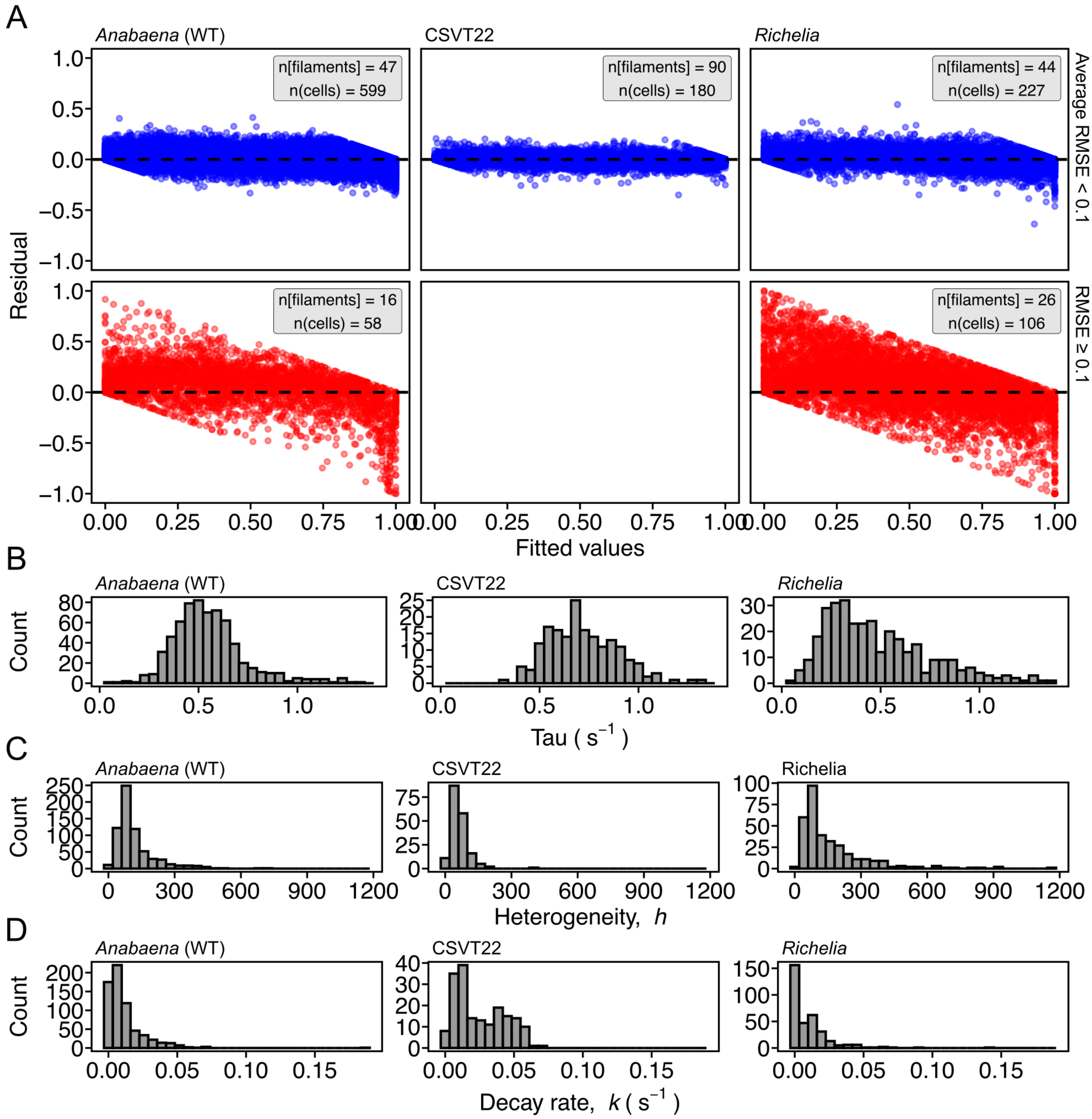

Figure S11 | StrExp model diagnostics and calculated model parameters (heterogeneity, tau and decay rate) for the strains *Anabaena* sp. PCC 7120, *Anabaena ΔfraC-ΔfraD* double mutant (CSVT22) and *Richelia rhizosoleniae* SC01. (**A**) Fited values of the StrExp functions against residuals. RMSE = Root Mean Squared Error. (**B**) Distribution of the decay time constant, Tau. (**C**) Distribution of the stretching parameter, *h*. (D) Distribution of the average decay rate, *k*.

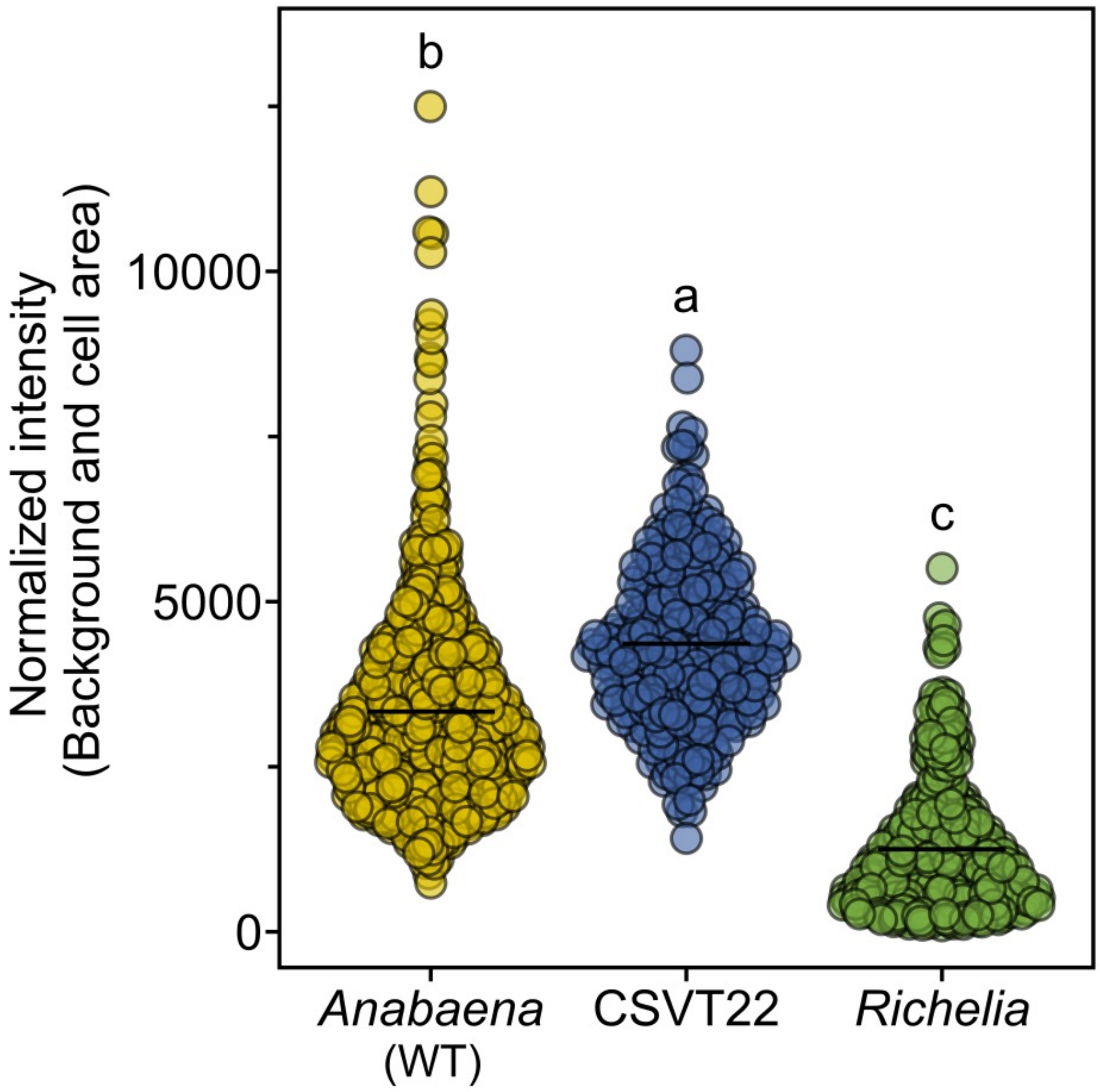

Figure S12 | Background and cell area normalized intensity distributions for each strain. Each point represents one cell. Different letters indicate significant differences between groups according to a Kruskal-Wallis test ($H[2] = 719$, $p < 0.001$, $n = 1473$ cells).

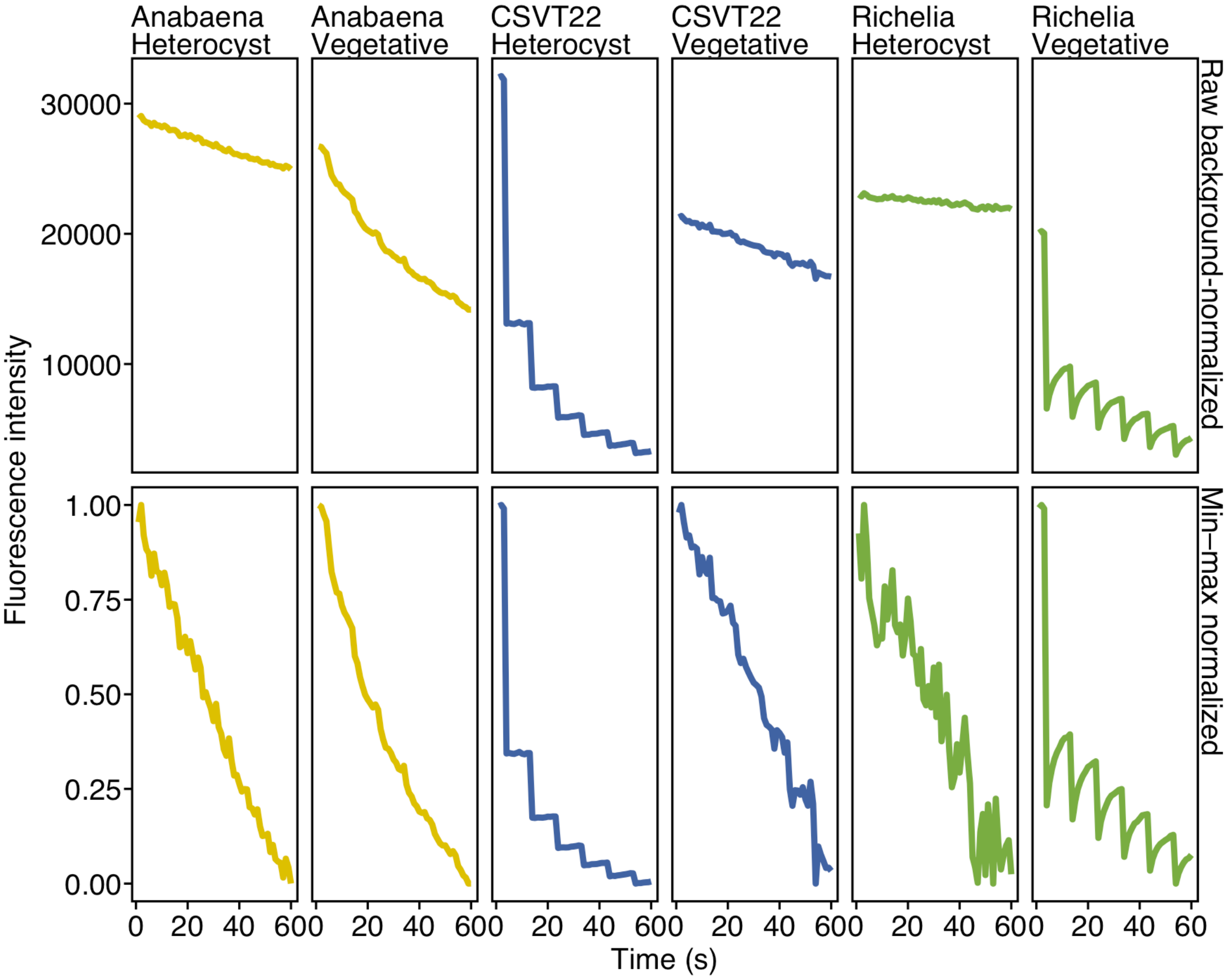

Figure S13 | Examples of non-normalized and normalized fluorescence intensity decay curves for each strain and bleached cell type.

Suppl. Table S1 | Summary of Fra and AmiC proteins encoded in the *Richelia* symbiont genomes. *Anabeana* sequences were used as queries in BlastP analyses (expect values indicated in parenthesis) to identify ORFS in the *Richellia* genomes: *Richelia* SC01 (RrhiSC01), endobionts: *R. intracellularis* (RintRC01), *R. euintracellularis* (ReuHH01). nd, not detected.

Anabaena	RrhiSC01	RintRC01	ReuHH01
**FraC, Alr2392**	2790723278 Ga0265390_13569 (4e-40)	RintRC_1395 (1e-51)	RINTHH_13270 (2e-44)
**FraD, Alr2393**	2790723277 Ga0265390_13568 (6e-125)	RintRC_1394 (e-106)	RINTHH_13260 (e-110)
**FraE, Alr2394**	2790723276 Ga0265390_13567 (4e-111)	RintRC_4487 (1e-60)	RINTHH_14600 (0.011)
**SepJ, Alr2338**	2790723170 Ga0265390_134818 (9e-110)	Incomplete RintRC_2278	RintHH_17990
AmiC1, Alr0092	2790720481 Ga0265390_11352 (0.0)	2622769080 Ga0055315_11862 (4e-66)	2580948732 RINTHH_21840 (0.0)
**AmiC2, Alr0093**	nd	nd	nd
**LytM regulator factor Alr3353**	2790719780 Ga0265390_10805 (9e-100)	2622771010 Ga0055315_14432 (2e-74)	RINTHH_10740 (5e-82)
**AmiC3, All1140**	2790719560 Ga0265390_106145 (e-158)	2622770008 Ga0055315_13045 (1e-153)	2580948076 RINTHH_15410 (9e-151)
