# Supplementary material for: Cell identity and filament architecture shape intercellular communication in the terminal heterocystous cyanobacterium Richelia rhizosoleniae SC01
Source: Appl Environ Microbiol. 2026 Sep 10;92(10):e00935-26. doi: 10.1128/aem.00935-26 (PMC13689106; doi:10.1128/aem.00935-26)
Supplement: Supplementary file 2 [file aem.00935-26-s0002.docx]

Supplemental methods

***Processing of data from TEM micrographs of murein sacculi from Richelia* SC01.** Septal disks which were fragmented or were significantly covered by material (e.g., porous material) were excluded from analyses. In total: 10 disks (22%) were included in the analyses; 24 (52%) disks were whole but significantly covered and excluded from analyses; 12 (26%) disks were fragmented. TEM micrographs (2048 by 2048 pixels, 8-bit) were processed by thresholding and binary conversion prior to analyzing particles with area limited to 1 x 10^-4^ to 0.01 µm^2^. Nanopores were identified by an automated analysis in ImageJ and subsequently filtered in R by the following selection criteria: circularity ≥ 0.4, convexity ≥ 0.7 and diameter < 100 nm. All images were manually checked for artifacts from the automated analysis prior to the filter step in R. For septal peptidoglycan disks, regions of interest (ROIs) were traced with the polygon tool: averaging 7 points/nanopore and 262 points/septal disks.

***Morphological measurements of cyanobacterial filaments***

**Supplementary Table 4**. Summary of criteria applied during morphological measurements of cyanobacterial filaments.

| Criterion | Rationale |
| --- | --- |
| Include filaments with ≥3 cells | Ensures reliable estimation of filament structure and avoids incomplete fragments |
| Exclude overlapping or clumped filaments | Prevents measurement errors due to unclear boundaries between cells |
| Count only filaments with whole filament visible within field of view | Ensures accurate measurement of filament length and cell number, preventing underestimation or bias caused by partially visible or truncated filaments |
| Count only mature heterocysts | Ensures comparability, as immature heterocysts differ morphologically and functionally |
| Count 100 filaments per replicate | Provides sufficient statistical power while remaining manageable |
|  |  |

***in silico analysis of DNA and protein sequences***

The search for sequences and data analysis of Fra and amidase proteins of *Richelia* strains was carried out using Integrated Microbial Genomes (IMG) of the Joint Genome Institute (JGI) and aligning the sequences of *Anabaena* sp. PCC 7120 and *Richelia* proteins with ClustalW (<https://www.ebi.ac.uk/jdispatcher/msa/clustalo?outfmt=clustal>) and default settings (Madeira et al., 2024).

***Selection of cell-distance ranges for FLIP analysis***

Two spatial ranges were selected for comparison of fluorescence decay kinetics in FLIP experiments. A distance of two cells from the bleached cell (d=2) was selected to capture near-junction exchange dynamics at the single-cell level and represents the maximum measurable distance in CSVT22 filaments, which had a length of 3 cells per filament, allowing inclusion of all three strains in this comparison. A distance of five cells (d=5) was selected to capture filament-level propagation of fluorescence loss and was restricted to *Anabaena* and *Richelia* SC01, which have sufficiently long filaments for reliable measurements at this distance. CSVT22 was excluded for the reason stated above. The 60-second experimental duration imposed a practical upper limit on detectable distances, as cells further than five cells from the bleached cell showed predominantly ambiguous decay profiles within this time frame, as reflected by the increasing proportion of very low *h* (heterogeneity parameter) values with distance (Fig. 5; Table 3; Wüstner et al., 2012).

***Assessment of model fit quality***

Model fit quality was evaluated using the p-values associated with estimated recovery rates (*R*). Cells for which the FRAP model could not be fitted were excluded from the dataset (CSVT22 heterocysts: n = 8; CSVT22 vegetative cells: n = 5; *Richelia* SC01 heterocysts: n = 1; *Richelia* SC01 vegetative cells: n = 3; *Anabaena*: n = 0). Fits with p ≥ 0.05 were considered non-significant. No FRAP profiles were excluded based on fit quality. Instead, cells with non-significant fits were retained in the dataset and assigned *R* = 0 s⁻¹, classifying them as non-communicating consistent with the criterion of *R* < 0.01 s⁻¹ (Arévalo et al., 2021; Flores et al., 2019). The proportion of non-significant fits per strain and cell type was as follows: *Anabaena* heterocysts 9.1% (2/22), *Anabaena* vegetative cells 6.1% (2/33), CSVT22 heterocysts 60.0% (18/30), CSVT22 vegetative cells 27.9% (17/61), *Richelia* SC01 heterocysts 20.0% (5/25), and *Richelia* SC01 vegetative cells 6.9% (4/58).

***References***

Arévalo, S., Nenninger, A., Nieves-Morión, M., Herrero, A., Mullineaux, C. W., & Flores, E. (2021). Coexistence of Communicating and Noncommunicating Cells in the Filamentous Cyanobacterium *Anabaena*. *mSphere*, *6*(1), e01091-20. <https://doi.org/10.1128/mSphere.01091-20>

Flores, E., Nieves-Morión, M., & Mullineaux, C. W. (2019). Cyanobacterial Septal Junctions: Properties and Regulation. *Life*, *9*(1), 1. <https://doi.org/10.3390/life9010001>

Madeira F, Madhusoodanan N, Lee J, et al. The EMBL-EBI Job Dispatcher sequence analysis tools framework in 2024. *Nucleic Acids Research*. 2024 Jul;52(W1):W521-W525. DOI: 10.1093/nar/gkae241. PMID: 38597606; PMCID: PMC11223882.

Wüstner, D., Solanko, L. M., Lund, F. W., Sage, D., Schroll, H. J., & Lomholt, M. A. (2012). Quantitative fluorescence loss in photobleaching for analysis of protein transport and aggregation. *BMC Bioinformatics*, *13*(1), 296. <https://doi.org/10.1186/1471-2105-13-296>
